# Polyploidy on islands – concerted evolution and gene loss amid chromosomal stasis

**DOI:** 10.1093/aob/mcac051

**Published:** 2022-04-07

**Authors:** Prashant Joshi, Helal Ansari, Rowan Dickson, Nicholas W Ellison, Cynthia Skema, Jennifer A Tate

**Affiliations:** School of Natural Sciences, Massey University, Palmerston North, New Zealand; AgResearch Grasslands Research Centre, Palmerston North, New Zealand; School of Natural Sciences, Massey University, Palmerston North, New Zealand; AgResearch Grasslands Research Centre, Palmerston North, New Zealand; School of Natural Sciences, Massey University, Palmerston North, New Zealand; Morris Arboretum of the University of Pennsylvania, Philadelphia, PA, USA; School of Natural Sciences, Massey University, Palmerston North, New Zealand

**Keywords:** 18S rDNA, chromosome number, concerted evolution, fluorescence *in situ* hybridization, GBSSI, gene fractionation, ITS, Malvaceae, *Plagianthus* alliance, polyploid, rDNA

## Abstract

**Background and Aims:**

Polyploidy is an important process that often generates genomic diversity within lineages, but it can also cause changes that result in loss of genomic material. Island lineages, while often polyploid, typically show chromosomal stasis but have not been investigated in detail regarding smaller-scale gene loss. Our aim was to investigate post-polyploidization genome dynamics in a chromosomally stable lineage of Malvaceae endemic to New Zealand.

**Methods:**

We determined chromosome numbers and used fluorescence *in situ* hybridization to localize 18S and 5S rDNA. Gene sequencing of 18S rDNA, the internal transcribed spacers (ITS) with intervening 5.8S rDNA, and a low-copy nuclear gene, *GBSSI-1*, was undertaken to determine if gene loss occurred in the New Zealand lineage following polyploidy.

**Key Results:**

The chromosome number for all species investigated was 2*n* = 42, with the first published report for the monotypic Australian genus *Asterotrichion*. The five species investigated all had two 5S rDNA signals localized interstitially on the long arm of one of the largest chromosome pairs. All species, except *Plagianthus regius*, had two 18S rDNA signals localized proximally on the short arm of one of the smallest chromosome pairs. *Plagianthus regius* had two additional 18S rDNA signals on a separate chromosome, giving a total of four. Sequencing of nuclear ribosomal 18S rDNA and the ITS cistron indicated loss of historical ribosomal repeats. Phylogenetic analysis of a low-copy nuclear gene, *GBSSI-1*, indicated that some lineages maintained three copies of the locus, while others have lost one or two copies.

**Conclusions:**

Although island endemic lineages show chromosomal stasis, with no additional changes in chromosome number, they may undergo smaller-scale processes of gene loss and concerted evolution ultimately leading to further genome restructuring and downsizing.

## INTRODUCTION

### Genome changes following polyploidy

Polyploidy, or whole genome duplication, is an important evolutionary process in flowering plants that has contributed to species diversification (Soltis and Soltis, 2016; Alix *et al.*, 2017; Kovařík *et al.*, 2017). Essentially, a polyploid has more than two sets of chromosomes, which is a relative condition in that the determination of being polyploid depends on comparison to a close relative and their number of chromosomes. The formation of polyploids can involve similar genomic combinations, leading to autopolyploids, or different genome combinations, leading to allopolyploids (Grant, 1981). Following polyploidy, especially allopolyploidization, which includes hybridization as part of the process, a number of genomic changes are common in plant lineages. The combination of divergent but compatible progenitor genomes in the allopolyploid triggers a number of responses akin to ‘genome shock’ (McClintock, 1984). These changes may involve larger-scale genome rearrangements and chromosomal translocations, homeologous recombination, activation of transposable elements, as well as smaller-scale gene losses and epigenetic modifications (e.g. as reviewed in Chen *et al.*, 2007; Wendel, 2015). The individual duplicated nuclear genes in the allopolyploid (homeologs) might maintain their original function, diverge in function, acquire a new function or be lost, leading to substantial genomic diversity post-polyploid formation (Lynch and Conery, 2000; Prince and Pickett, 2002).

The responses to allopolyploidy are varied and differ across lineages and may depend upon the degree of divergence between the progenitor genomes (Paun *et al*., 2009). Over time, the myriad genomic changes in polyploids lead to a diploid-like state, through either chromosomal diploidization or gene fractionation and diploidization (Li *et al.*, 2021). In the former scenario, meiotic chromosomal pairing ultimately mimics former diploid genomes, while in the latter, small-scale changes accumulate such that polyploid species return to a diploid-like state (Wendel *et al.*, 2018; Li *et al.*, 2021). Broad comparative analyses of nuclear gene loss across flowering plant polyploids of varying ages indicate that particular categories of genes are alternatively lost or maintained in duplicate following polyploidy (Paterson *et al.*, 2006; Li *et al.*, 2016).

**Fig. 1.**
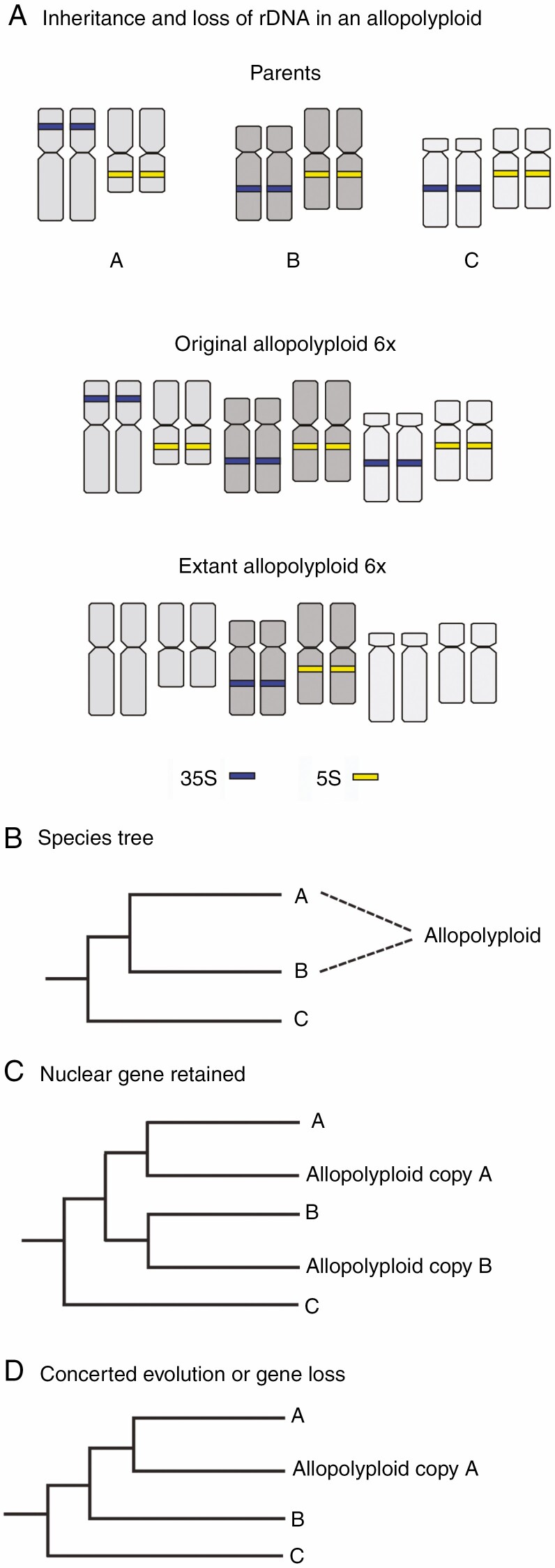
rDNA evolution and phylogenetic outcomes in an allopolyploid. (A) Allopolyploids are expected to inherit rDNA loci from each parent; in this case a hexaploid (6*x*) will inherit a 35S and a 5S locus from each of its three parents. Initially it will be expected to maintain each of those sites upon allopolyploid formation. Over time, some of these loci may be lost via concerted evolution and the original parental signatures can be lost from the genome. (B) Allopolyploids show reticulate evolution with two or more lineages merging together into the new allopolyploid lineage. Here an allopolyploid lineage forms from Species A and Species B creating a new allopolyploid. (C) As each nuclear gene is duplicated in an allopolyploid (in this case a tetraploid), the expectation is that the polyploid would retain a gene copy (homeologue) from each of its parental lineages. In the phylogeny these gene copies are informative about the lineage that contributed to the allopolyploid genome. (D) If concerted evolution or gene loss occurs for the locus under study, then only one parental locus will appear in the phylogeny.

Among the regions that show rapid post-polyploidization changes are the nuclear ribosomal DNA (rDNA loci (Weiss-Schneeweiss *et al.*, 2008; Weiss-Schneeweiss and Schneeweiss, 2013). In plants, rDNA regions are typically organized into separate 5S and 35S (18S–5.8S–26S) rDNA regions that exist as tandemly arranged units with hundreds or thousands of copies (Heslop-Harrison and Schwarzacher, 2011; Garcia *et al.*, 2017). Concerted evolution acts to homogenize these multiple copies within the active cistron (Eickbush and Eickbush, 2007). As with other gene regions, the null expectation is that following formation, polyploids would show increasing numbers of rDNA loci as ploidal level increases (Fig. 1A, e.g. Lattier *et al.*, 2019; Qu *et al.*, 2021). In some polyploids where the numbers of loci are additive of the parents, one inherited set may be transcriptionally suppressed (nucleolar dominance) and the 18S–26S rDNA locus remains visibly condensed (Pikaard, 1999; Matyášek *et al.*, 2007; Ansari *et al.*, 2008). Additionally, the loss of rDNA loci via concerted evolution in polyploids is a common occurrence, sometimes occurring at the early stages following formation of polyploids or over longer evolutionary timescales (Fig. 1; Wendel *et al.*, 1995; Kovařík *et al.*, 2005, 2008; Williams *et al.*, 2012; Vaio *et al.*, 2019). Such loss means that the parental origins of polyploids can often be obscured by the uniparental retention of rDNA loci (Alvarez and Wendel, 2003). In phylogenetic studies, this situation can be particularly problematic for recovering polyploid origins where duplicated nuclear gene copies would otherwise convey the parental origin of the allopolyploid (Fig. 1B–D) (see also Rothfels, 2020).

### Polyploidy on islands

Islands are natural laboratories for evolutionary studies (Carlquist, 1974). Island taxa may reflect the properties of their continental relatives or may display new traits not apparent in their continental or source-related lineages (Crawford *et al.*, 2009; Soltis *et al.*, 2014; Crawford and Archibald, 2017). Polyploidy has been noted to occur in island taxa to varying degrees, with a recent global study (Rice *et al.*, 2019) noting high proportions of polyploids on islands. The frequent occurrence of polyploidy on islands may be a consequence of dispersal traits of polyploids or of lineages that tend to establish on islands (Linder and Barker, 2014; Vargas *et al.*, 2015). Once established on the island, however, many taxa show chromosomal stasis in which there are no further changes in chromosome numbers or ploidal levels (Stuessy and Crawford, 1998). These lineages typically arrive on the island as polyploids and do not further diversify in their ploidal levels (Carr, 1998). These trends typify some, but not all, polyploid lineages on islands (Meudt *et al.*, 2021). Given the wider trend that most polyploids undergo gene fractionation and/or genome downsizing, might there be smaller-scale genomic changes in these lineages that ultimately might lead to additional chromosomal diversity?

### Polyploidy on islands – a case study from New Zealand Malvaceae

The native New Zealand flora has high levels of species endemicity (~80 %; de Lange *et al.*, 2018; Schönberger *et al.*, 2019) and while hybridization and polyploidy are common features of the native flora (Morgan-Richards *et al.*, 2009; Meudt *et al.*, 2021), for most taxa their origins are not known (Murray and de Lange, 2011). Some phylogenetic studies have revealed that the closest relatives to New Zealand polyploid taxa are overseas rather than New Zealand congeners. For example, the seven polyploid species of *Plantago* (Plantaginaceae) in New Zealand represent two lineages that are more closely related to separate Australian diploid lineages than to New Zealand species (Tay *et al.*, 2010). In other cases, the New Zealand species of a genus are all polyploids derived from an overseas relative [e.g. *Ourisia* (Plantaginaceae), Meudt and Simpson (2006)] and may have additional polyploid species derived from New Zealand taxa [e.g. *Melicytus* (Violaceae), Mitchell *et al.* (2009)]. To date, few studies have examined the genomic consequences of polyploidization in New Zealand plant taxa. Two studies thus far have found evidence of genome downsizing in polyploid species as compared to their relatives [e.g. *Veronica* (Plantaginaceae), Meudt *et al.* (2015) and *Plantago*, Wong and Murray (2012)], but on the whole we know very little about post-polyploidization genome dynamics in native plant lineages in New Zealand. Further, studying natural polyploids that have formed over varying evolutionary timescales offers valuable insight into the modes of genome changes following polyploidization (Mandáková *et al.*, 2010*b*; Soltis and Soltis, 2016).

To further our understanding of genome consequences following polyploidization in native New Zealand (NZ) plant lineages, we undertook a phylogenetic and molecular cytogenetic study of closely related genera in the Malvaceae (subfamily Malvoideae, tribe Malveae), which are Australasian members of the *Plagianthus* alliance (Bates, 1968; Tate *et al.*, 2005). For this group, previous phylogenetic analyses showed that *Hoheria* (seven species, NZ endemics) is the sister group to a clade of *Plagianthus* (two species, NZ endemics), *Asterotrichion* (monotypic genus endemic to Tasmania) and *Gynatrix* (two species, endemic to mainland Australia) (Tate *et al.*, 2005; Wagstaff *et al.*, 2010). The closest relatives to this group include *Lawrencia* (~20 species) from Australia and species of *Sida* and *Ripariosida*, from Australia and North America, respectively. Published chromosome counts for *Hoheria* and *Plagianthus* indicate that all species in New Zealand are 2*n* = 42 (Bates and Blanchard, 1970; Groves and Hair, 1971). These taxa are probably hexaploids given that the chromosome number available for the most closely related species outside of this group is *n* = 14 [*Ripariosida hermaphrodita* from eastern North America (Davie, 1935)], and a base of *x* = 7 or 8 is suggested for the tribe Malveae as a whole (Bates and Blanchard, 1970; Tate *et al.*, 2005). Published chromosome counts are not available for *Asterotrichion* or *Gynatrix*, nor are they available for *Lawrencia*. The aims of the present study were to determine chromosome counts, characterize rDNA loci using fluorescence *in situ* hybridization (FISH), and investigate post-polyploidization effects on two nuclear loci via gene sequencing for this group. Given the stability in chromosome number of the New Zealand taxa, we hypothesized that chromosomal stasis characterizes the group, but that smaller-scale gene losses may have occurred.

## MATERIALS AND METHODS

### Plant material

Plants for cytogenetic analysis were grown from seed. *Asterotrichion discolor* seeds were obtained from B&T World Seeds (http://b-and-t-world-seeds.com). Seeds of *Hoheria angustifolia*, *H. populnea* and *Plagianthus regius* were collected from trees growing on the campus at Massey University, Palmerston North, New Zealand. Seeds of *P. divaricatus* were collected from wild plants growing in the Rangitikei coastal estuary at Tangimoana, New Zealand. Vouchers of these plants are deposited in the Dame Ella Campbell Herbarium (MPN) at Massey University. In addition to these plants, other plant material for gene sequencing was obtained from field-collected plants or from herbarium specimens. For the gene sequencing we included a broader sampling of related taxa in the Malveae based on previous phylogenetic studies (e.g. Tate *et al.*, 2005). The Supplementary Data include the collection details of the plants used.

### Chromosome preparation and in situ hybridization

Seeds from all species were germinated in Petri dishes at room temperature. Seedlings were transferred into pots and allowed to establish in a glasshouse. Actively growing root tips from established plants were harvested and treated with 4 mm hydroxyquinoline solution for 2 h at room temperature and then for 6 h at 4 °C. These were then fixed in 3 : 1 methanol–acetic acid and stored at 4 °C until use. Somatic chromosome preparations from the fixed root tips were obtained according to the flame drying technique described by Ansari *et al.* (2016). Briefly, the fixed root tips were macerated in 2 % (w/v) cellulase (1.6 % cellulase Calbiochem 515883 + 0.4 % cellulase Onozuka R-10) in citrate buffer at pH 4.8 and 20 % (v/v) pectinase (from *Aspergillus niger* in 40 % glycerol; Sigma P-0690) for 45–55 min at 37 °C. On a clean glass slide, meristematic tissue from root tips was gently extruded from surrounding tissues in a droplet of phosphate buffer under a stereomicroscope. The meristematic tissue was dissociated into a cell suspension using fine needles. Thereafter, one drop of 48 % acetic acid was gently placed on the cell suspension. After 2 min of incubation at room temperature, two or three drops of chilled methanol–acetic acid (3 : 1) fixative (stored at −20 °C) was gently placed on the cell suspension and quickly flame-dried. Slides were screened using phase contrast optics to assess the quality of cytological preparations before they were subjected to FISH procedures.

For FISH, 18S and 5S rDNA probes from *Trifolium repens* (GenBank accessions AF071069 and AF072692) were used (Ansari *et al.*, 1999). These rDNA probes were labelled with Fluor-X-dCTP and Cy3-dCTP (GE Healthcare, NZ), respectively, by nick translation according to the manufacturer’s specifications. Double-target FISH was carried out on RNase-treated denatured chromosomal preparations. *In situ* hybridization of 5S and 18S rDNA, and post-hybridization washing was processed exactly as described by Ansari *et al.* (1999). Chromosomes were counterstained with DAPI (0.5 µg mL^–1^ in McIlvaine’s buffer, pH 7) for 5 min before mounting in Vectashield (Vector Laboratories). At least ten cells were observed per sample and photomicrographs were acquired on a Microphot-S microscope (Nikon, Japan) fitted with an AxioCam MRm CCD camera (Carl Zeiss, Germany) using ISIS imaging software (MetaSystems, Germany).

### Primer design for gene sequencing

Three nuclear regions were amplified to determine if loss of ribosomal repeats or nuclear genes had occurred in these lineages. First, we amplified a large (~1.62 kb) portion of the 18S rDNA gene by designing primers from aligned sequences from Malvaceae available in GenBank (*Gossypium hirsutum*: L24145, *Callirhoe involucrata*: KT459179, *Sphaeralcea coccinea*: KT459212, *Abelmoschus esculentus*: MG792348). We verified conserved regions in the 18S rDNA for primer design by also aligning these sequences to *Trifolium repens* (AF071069). Amplification primers were located ~150 bp (150F: 5′-GAGCTAATACGTGCAACAAACC) and ~1760 bp (1760R: 5′-CCTTGTTACGACTTCTCCTTCC) downstream of the start of the 18S rDNA gene, respectively. We also designed internal primers at ~1000 bp to aid in sequencing – these two primers were slightly overlapping reverse complements of one another (1000F: 5′-CCGTCCTAGTCTCAACCATAAAC and 1000R: 5′-TGGTTGAGACTAGGACGGTATC).

Second, we amplified the internal transcribed spacer (ITS) region, including the 5.8S gene, using primers as described in Tate *et al.* (2005). Third, we amplified a low-copy nuclear gene, Granule-bound starch synthase (*GBSSI* or *waxy*), to represent a locus potentially not subjected to concerted evolution (Mason-Gamer *et al.*, 1998). *GBSSI* was amplified with primers placed within exons 1 (WAXY1F) and 9 (WAXY9R) as designed by Evans *et al.* (2000) for studies in Rosaceae. Three copies of *GBSSI* are present in Malvaceae (R. Small, unpubl. data), but only a single locus (*GBSSI-1*) is amplified in tribe Malveae using the above primers and PCR conditions as described below (Small, 2004).

### DNA extraction, PCR, cloning and Sanger sequencing

DNA was extracted from silica gel-dried leaves from field-collected material or herbarium specimens (Supplementary Data) following a modified CTAB protocol (Doyle and Doyle, 1987) or using the Plant/Seed DNA MiniPrep kit (Zymo Research, Irvine, CA, USA) following the manufacturer’s instructions. Extracted DNA was run on a 1 % agarose gel, stained with ethidium bromide and visualized on a UV transilluminator.

PCR amplification of 18S rDNA and the ITS region followed Tate *et al.* (2005) and utilized a touchdown PCR programme consisting of 95 °C for 5 min, then five cycles of 95 °C for 1 min, 53 °C for 1 min and 72 °C for 2 min, followed by 44 cycles of 95 °C for 1 min, 48 °C for 1 min and 72 °C for 2 min, and a final 72 °C extension for 7 min. For *GBSSI*, PCRs and cycling conditions were as described by Small (2004), although scaled down to 13 μL reactions; TaKaRa ExTaq polymerase (TaKaRa, Otsu, Shiga, Japan) was utilized. Because some of the DNA extractions from herbarium collections were degraded, a smaller portion of *GBSSI-1* was amplified using a new primer designed within exon 2 (WAXY2Fb – 5′-GACDGTDTCTCCTMGGTACGAS) along with WAXY9R, and a reduced annealing temperature of 55 °C.

Where multiple bands were apparent in the visualized PCR product, cloning was used to separate each band for sequencing. The TOPO-TA Cloning Kit for Sequencing (Invitrogen, Carlsbad, CA, USA) was utilized, with some modifications to the manufacturer’s protocol. Colonies were screened for the inserts of interest by colony PCR using NEB Taq polymerase (New England Biolabs, Ipswich, MA, USA) and either the T3/T7 or M13F/M13R primer pairs targeting vector sequences flanking the insert. Where possible, at least three products of differing sizes were sequenced to screen for potential homeologous copies. Permission to clone from the native New Zealand species was granted by Tanenuiaranga Manawatu Inc.

PCR products were prepared for sequencing by an ExoSap digest (Exonuclease I and shrimp alkaline phosphatase; Fermentas, Glen Burnie, MD, USA). Sanger sequencing utilized the BigDye Terminator v3.1 Cycle Sequencing Kit (Applied Biosystems, Foster City, CA, USA), and cleaned products were run on an ABI3730 DNA Analyser (Applied Biosystems) at the Massey Genome Service (Palmerston North, NZ).

### Sequence editing and phylogenetic analyses

Forward and reverse sequences were assembled into contigs and edited in Geneious v.9.1.8 (Biomatters, Auckland, NZ). For 18S rDNA and the ITS region, the chromatograms were visually inspected for evidence of polymorphisms in the sequence, which would indicate multiple sequence types. For *GBSSI-1*, the sequences were aligned to a published *Hibiscus moscheutos GBSSI* sequence (GenBank: AY341414) to identify exon–intron boundaries. Recombinant cloned sequences were detected using RDP v.3.44 (Martin *et al.*, 2010). Initial analyses included multiple sequences from each species that were largely identical and probably represented allelic variants; if these alleles clustered together with strong statistical support, then a single variant was included in subsequent analyses.

Because there was little sequence variation within the 18S rDNA region, a phylogenetic analysis was not conducted for this locus. The aligned 18S rDNA sequences are presented in the Supplementary Data. For the ITS and *GBSSI* datasets, phylogenetic analyses employed maximum likelihood in RaxML v.7.2.8 with GTR-CAT as the selected model and rapid bootstrapping (Stamatakis, 2006) and Bayesian analysis conducted in MrBayes v.3.2 (Ronquist *et al.*, 2012) following model selection in MrModeltest v.2.4 (Nylander, 2004). Data partitions were defined for coding and non-coding regions as follows, ITS: ITS1 and ITS2 – GTR + I, 5.8S – K80; GBSSI: exon 2 – JC + I, intron 3 – HKY + I, exons 3 and 4 – K80, intron 4 – HKY + G, exon 5 and intron 5 – SYM. MrBayes was run for least 20 million generations or until the average standard deviation of the split frequencies reached 0.001. Trees corresponding to the burn-in period (the first 25 %) were discarded and a majority-rule consensus was constructed from the remaining trees. To examine reticulate evolutionary relationships within this group, we also used Splitstree (Huson and Bryant, 2006) on the *GBSSI-1* data set to reconstruct a Neighbor-net splitsgraph. *Lecanophora chubutensis* was designated as the outgroup in all phylogenetic analyses (Tate *et al.*, 2005). Newly generated sequences were submitted to GenBank (Supplementary Data).

## RESULTS

### Chromosome counts and rDNA FISH

**Fig. 2.**
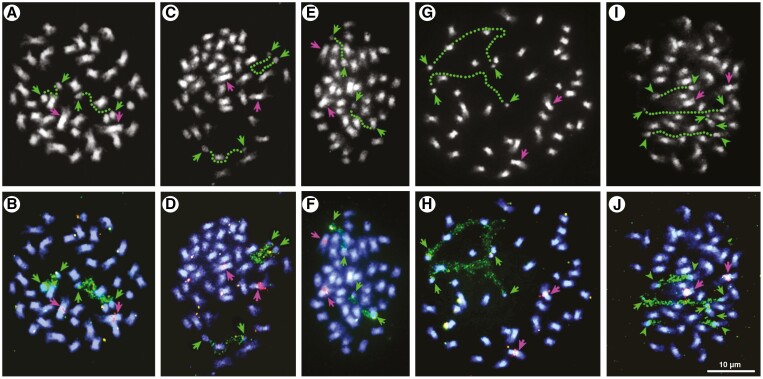
Distribution of 5S and 18S–26S rDNA sequences on somatic chromosomes of five species of the *Plagianthus* alliance. DAPI-stained (greyscale) metaphase cell of *Asterotrichion discolor* (A), *Hoheria populnea* (C), *H. angustifolia* (E), *Plagianthus divaricatus* (G) and *P. regiu*s (I) and the same cells with FISH signals of 5S (red) and 18S–26S (green) rDNA sequences in B, D, F, H and J, respectively, where chromosomes are counterstained with DAPI (blue). Decondensed nucleolar organizing regions (NORs) visible in FISH preparations are denoted with green dotted lines in A, C, E, G and I. Green arrows denote condensed parts of NOR-carrying chromosomes while pink arrows indicate chromosomes mapping 5S rDNA loci.

The diploid chromosome number of all three genera under study was 2*n* = 42 (Fig. 2; Table 1). Notably, the same number was found for *Asterotrichion discolor*, which was investigated here for the first time (Fig. 2A, B). Chromosome morphology among all taxa was similar (Fig. 2). In all five taxa, the biarmed chromosomes showed a gradual transition in size.

**Table 1.** Diploid (mitotic) chromosome number and distribution of 5S and 18S rDNA loci based on fluorescence *in situ* hybridization.

Species	2*n* number	No. of 5S signals	No. of 18S signals
*Asterotrichion discolor*	2*n* = 42	2	2
*Plagianthus regius*	2*n* = 42	2	4
*Plagianthus divaricatus*	2*n* = 42	2	2
*Hoheria populnea*	2*n* = 42	2	2
*Hoheria angustifolia*	2*n* = 42	2	2

Molecular cytogenetic analysis revealed similarities among *Asterotrichion discolor* (Fig. 2A, B; Table 1), *Hoheria populnea* (Fig. 2C, D), *H. angustifolia* (Fig. 2E, F) and *Plagianthus divaricatus* (Fig. 2G, H). Each displayed two 18S rDNA signals and two 5S rDNA signals, which mapped to two separate chromosome pairs. The 18S rDNA signals were localized proximally on the short arm of one of the smallest chromosome pairs, whereas 5S rDNA signals were mapped interstitially on the long arm of one of the three largest chromosome pairs. A different condition was observed in *Plagianthus regius*, which showed four signals of 18S rDNA and two signals of 5S rDNA (Fig. 2I, J; Table 1). The two additional signals of 18S rDNA were mapped onto a separate chromosome pair, again among the smaller ones. In *P. regius*, the 5S rDNA signals were again localized interstitially on the long arm of one of the largest chromosome pairs. In all FISH experiments, 18S rDNA loci were found to be decondensed, revealing its active nature.

### 18S rDNA sequences

An 18S rDNA alignment of ~1590 bp was generated (Supplementary Data) for 21 individuals (including two outgroups). None of the 18S rDNA sequences showed evidence of sequence polymorphisms (i.e. multiple peaks in the chromatogram), indicating that the 18S rDNA locus has undergone concerted evolution. Sequences of *A. discolor*, *Gynatrix pulchella,* and species of *Hoheria* and *Plagianthus* were identical with the exception of one polymorphism in *P. divaricatus* and one in *G. pulchella* (Supplementary Data). The other potentially phylogenetically informative sites occurred within *Lawrencia* species or *Lecanophora chubutensis*, but none of these showed evidence of site polymorphisms.

### ITS sequencing and phylogeny reconstruction

**Fig. 3.**
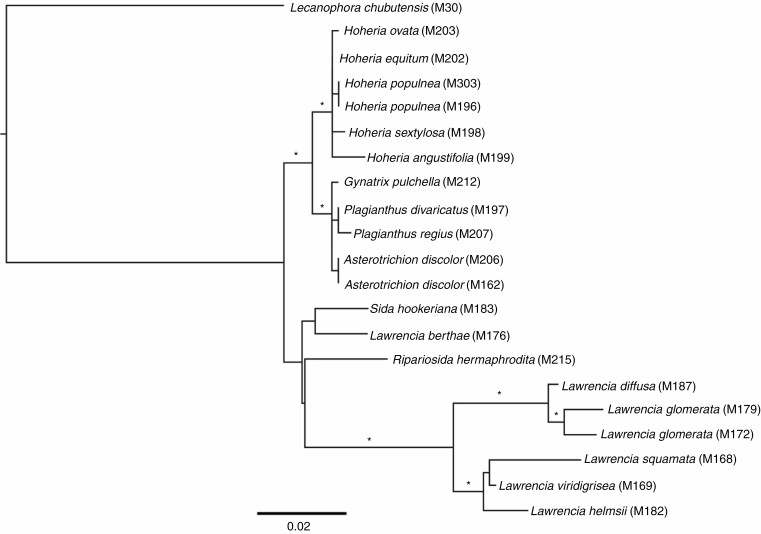
Phylogenetic tree derived from RAxML and Bayesian analysis of nuclear ribosomal ITS sequences for the *Plagianthus* alliance. *Lecanophora chubutensis* was the designated outgroup. An asterisk indicates clades with > 80 % maximum-likelihood bootstrap support values and > 0.95 Bayesian posterior probabilities.

ITS sequences were generated for 20 individuals, and one sequence (*Lecanophora chubutensis*) was utilized from a previous study (Tate *et al.*, 2005). A few sequence polymorphisms were evident in the chromatograms within the non-coding spacer regions (ITS1: *H. populnea* M303, R [A/G] at position 22 and M196, K [G/T] at position 67 and M [A/C] at position 182; *H. equitum* M200 S [C/G] at position 155. ITS2: *H. ovata* M203 W [A/T] at position 578 and Y [C/T] at position 682; *H. angustifolia* M199 Y [C/T] at position 673). There were no polymorphisms within the 5.8S gene. The aligned length of the entire ITS region (ITS1, 5.8S gene, ITS2) was 697 bp. The relationships returned in the ITS tree (Fig. 3) are similar to those based on previous studies (Tate *et al.*, 2005): *Hoheria* is sister to a clade consisting of *A. discolor*, *G. pulchella* and *Plagianthus* species. A second clade includes most of *Lawrencia* forming a single clade with another clade that includes *Sida hookeriana*, *Lawrencia berthae* and *Ripariosida hermaphrodita*.

### GBSSI-1 sequencing and phylogeny reconstruction

**Fig. 4.**
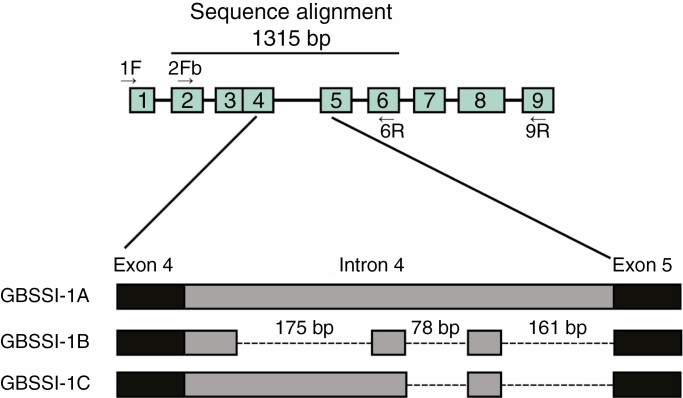
Schematic of diagnostic indel patterning of intron 4 of *GBSSI-1* for the *Plagianthus* alliance. The pattern proved to be diagnostic for each copy of *GBSSI-1* retrieved from the group under study and was utilized to assign putative homology amongst cloned sequences.

Partial *GBSSI* sequences were obtained, most spanning from exon 2 to exon 5; those with homology to *GBSSI-1* could be ascertained via analysis of intron 3, which is absent in this copy within the tribe Malveae (R. Small, pers. comm.). A single copy of *GBSSI-1* was retrieved from the outgroup *Lecanophora chubutensis*, *R. hermaphrodita*, and all *Lawrencia* representatives except *Lawrencia berthae* (Table 2). Multiple copies were detected in species of *Hoheria* and *Plagianthus*, as well as *S. hookeriana*, *Lawrencia berthae*, *Asterotrichion discolor* and *G. pulchella*; appreciable size differences allowed these to be distinguished via gel electrophoresis. Three sequences (from *P. regius*, *H. sexstylosa* and *Lawrencia berthae*) were identified as being of recombinant nature, although it is likely that these represent an artefact of PCR; these were found to significantly alter phylogenetic inference and were therefore excluded from the final analyses. A total of 38 sequences were included to construct an alignment of 1314 bp, with 183 of these characters variable and phylogenetically informative. The aligned lengths of the sequenced regions were exon 2 (partial): 61 bp, intron 2: 96 bp, exon 3: 99 bp, exon 4: 90 bp, intron 4: 893 bp, exon 5: 64 bp; intron 5 (partial): 11 bp (Fig. 4). Among the three *GBSSI-1* copies, exon lengths were not variable. Intron 4 was highly variable among copies and could be used to assign putative homology among clones from different taxa based on distinctive indel patterning, as visualized in Fig. 4. Table 2 details the different copies recovered from the sequenced samples.

**Table 2.** *GBSSI-1* copies retrieved from species of the *Plagianthus* alliance

Taxon	Code	*GBSSI*-*1A*	*GBSSI*-*1B*	*GBSSI*-*1C*	*GBSSI*-*1*^†^
*Asterotrichion discolor*	M206	x			
*Asterotrichion discolor*	M211	x	x		
*Asterotrichion discolor*	M306	x	x		
*Gynatrix pulchella*	M212	x	x		
*Hoheria angustifolia*	M199	x	x	x	
*Hoheria equitum*	M202	x	x	x	
*Hoheria ovata*	M203	x	x	x	
*Hoheria populnea*	M196	x	x		
*Hoheria populnea*	M303	x		x	
*Hoheria sexstylosa*	M198	*	x	x	
*Lawrencia berthae*	M176	x	x	x	
*Lawrencia diffusa*	M187			x	
*Lawrencia glomerata*	M172			x	
*Lawrencia glomerata*	M179			x	
*Lawrencia helmsii*	M182			x	
*Lawrencia squamata*	M168			x	
*Lawrencia viridigrisea*	M169			x	
*Lecanophora chubutensis*	M30				x
*Plagianthus divaricatus*	M197	x	x		
*Plagianthus regius*	M207	*	x		
*Ripariosida hermaphrodita*	M215				x
*Sida hookeriana*	M183		x	x	

x, copy was retrieved from this taxon.

*Copy was retrieved but was found to be of recombinant nature.

^†^Copy could not be delimited.

**Fig. 5.**
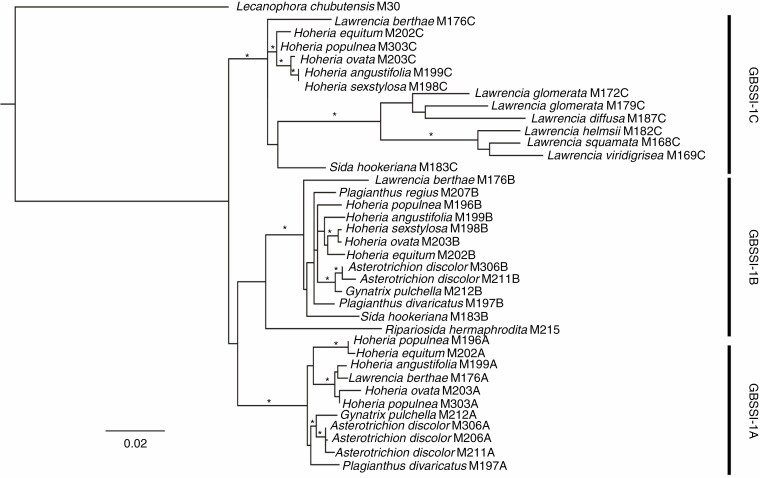
Phylogenetic tree derived from RAxML and Bayesian analysis of granule-bound starch synthase (*GBSSI-1*) sequences for the *Plagianthus* alliance. *Lecanophora chubutensis* was the designated outgroup. An asterisk indicates clades with > 80 % maximum-likelihood bootstrap support values and > 0.95 Bayesian posterior probabilities.

**Fig. 6.**
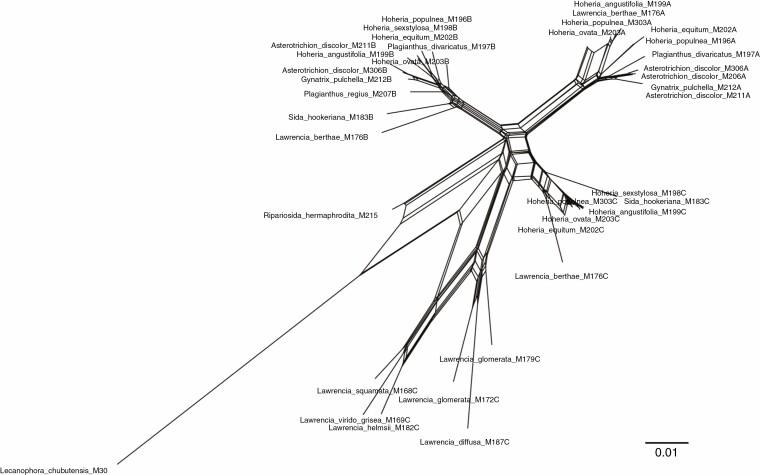
Neighbor-net splitsgraph of *GBSSI-1* sequences based on uncorrected P-distances for the *Plagianthus* alliance.

Three main clades were reconstructed in the RAxML and Bayesian analyses (Fig. 5), corresponding to the different copies of *GBSSI-1*, hereafter referred to as *GBSSI-1A*, *GBSSI-1B* and *GBSSI-1C*, as delimited in Fig. 4. Support values for each of these gene clades was high (excluding outgroups). Within the *GBSSI-1C* clade, *Lawrencia* (excluding *Lawrencia berthae*) was reconstructed as a separate strongly supported clade (Fig. 5). The sequences recovered from the *Lawrencia* taxa with a single copy are apparent as *GBSSI-1C* orthologues. *Sida hookeriana* was included within this clade but without support for its relationship to the other taxa. *Ripariosida hermaphrodita* was reconstructed as sister to the *GBSSI-1B* clade, again without support (Fig. 5). This clade comprised primarily *A. discolor*, *G. pulchella*, *Hoheria* and *Plagianthus* sequences along with *Lawrencia berthae* and *S. hookeriana GBSSI-1B* copies. The *GBSSI-1A* clade included sequences of *A. discolor*, *G. pulchella*, *Hoheria* and *Plagianthus* species, and *Lawrencia berthae*. The Neighbor-net splitsgraph recovered the same groups (Fig. 6) but also highlighted many reticulate events.

## Discussion

### Chromosomal stasis and rDNA evolution in the Plagianthus alliance

Naturally occurring allopolyploids and synthetic interspecific hybrid polyploids generally exhibit various genomic changes, which also include changes at the chromosomal level when compared with their progenitor species. Amplification, loss or expression dominance of 18S–26S rDNA loci (generally referred to as nucleolar dominance; Pikaard, 1999) from one sub-genome have been frequently reported. Although the recently formed allotetraploids *Tragopogon mirus* and *T. miscellus* (Asteraceae) show an additive number of rDNA loci for their ploidy level when compared with their diploid progenitors, nucleolar dominance in the form of a decondensed (active) 18S–26S rDNA locus from one ancestral parent and highly condensed (silenced) locus from the other parent has been demonstrated (Matyášek *et al.*, 2007). An identical status of uniparental nucleolar dominance has also been reported in allotetraploid *Trifolium dubium* (Fabaceae) where 18S–26S rDNA locus from the ancestral *T. campestre*-derived subgenome is decondensed (transcriptionally active) while that from *T. micranthum* remains highly condensed throughout the cell cycle (Ansari *et al.*, 2008). However, the allotetraploid forage legume, *Trifolium repens*, evolutionarily lost the 18S–26S rDNA locus from the ancestral parent sub-genome of *T. pallescens*, while that from the *T. occidentale*-derived sub-genome was preserved and remains active (Williams *et al.*, 2012).

The chromosome count for the taxa studied in the *Plagianthus* alliance was consistently 2*n* = 42 (Fig. 2), in agreement with previous counts for *Hoheria* and *Plagianthus* (Bates and Blanchard, 1970; Groves and Hair, 1971). The new count of 2*n* = 42 for *Asterotrichion* aligns this taxon with those from New Zealand, reflecting the close phylogenetic relationship among these genera (Tate *et al.*, 2005; Wagstaff *et al.*, 2010). The morphology of the chromosomes appeared similar among all the species studied, with mainly meta/submetacentric chromosomes with gradual transition in the size of the chromosomes (symmetric karyotype). The main difference among the five taxa studied was the additional 18S rDNA signal in *P*. *regius* (Fig. 2). At present, we do not have an explanation for this extra signal in *P. regius*, which could represent a historical locus retained from the polyploid event or could be a nucleolar organizing region (NOR) of separate origin. The 35S rDNA region in particular is known to be dynamic in polyploids and to undergo changes that can increase or decrease their number (Weiss-Schneeweiss *et al.*, 2008; Weiss-Schneeweiss and Schneeweiss, 2013). Notably, Schubert and Wobus (1985) found that NORs in *Allium* behaved like transposons and relocated to different chromosomes. The lack of positional shifts of the 5S and 18S rDNA loci and the relative consistency in the number of 5S rDNA as compared to 18S rDNA within this group is consistent with other observations in angiosperms (Roa and Guerra, 2015). More specifically, the interstitial position of the 5S rDNA loci is typical for angiosperms (Garcia *et al.*, 2017), while the proximal position of the 18S rDNA is more unusual. Garcia *et al.* (2017) note that where multiple 35S rDNA loci exist across plant lineages, the position of these loci tends to vary more than when one locus is present. Vaio *et al.* (2019) recently showed variability of 35S rDNA sites in *Paspalum*, representing both gains and losses of sites among closely related polyploid species, and even within a single species. Similar to other groups, we also find different dynamics between the 35S and 5S rDNA repeats, with more variability among the 35S loci (Hanson *et al.*, 1996).

The closest relative to the Australasian lineage with a published chromosome count is *R. hermaphrodita* with *n* = 14 (Davie, 1935), and a base of *x* = 7 or 8 has been suggested for the tribe Malveae (Bates and Blanchard, 1970; Tate *et al.*, 2005). Thus, assuming a base of *x* = 7, the chromosome counts of 2*n* = 42 indicate a hexaploid level for the taxa studied here. As such, in the somatic FISH results, we would expect to see three pairs of rDNA loci for both 5S and 18S rDNA. As only one pair was detected for most individuals, we infer loss of rDNA in this lineage since polyploid formation. The additional 18S rDNA signal in *P. regius* could reflect either retention of an original locus following the polyploidization event or gain of a novel locus. Like *Paspalum* (Vaio *et al.*, 2019), the two *Plagianthus* species may be undergoing independent diploidization (gene fractionation) processes, which are reflected in their differing 18S rDNA signals.

Interestingly, the species studied here show not only the same chromosome number, but also similar chromosome morphology and hence it is proposed that they also exhibit gross karyotypic stasis. Chromosomal stasis, where no change of chromosome number occurs, has historically been considered to typify island endemics (Stuessy and Crawford, 1998). However, polyploidy is known to be important in the establishment and diversification of island taxa (Meudt *et al.*, 2021) and while there may not (yet) be detected changes in chromosome number or structure among the taxa studied here, we do observe a number of gene losses (i.e. loss of rDNA and GBSSI loci) that may ultimately lead to more drastic diploidization within these lineages. Similarly, in *Pachycladon* (Brassicaceae), an allopolyploid derived from distinct crucifer lineages (Joly *et al.*, 2009), the polyploid species found in New Zealand all have the same chromosome number (i.e. chromosomal stasis) and karyotype, but they differ in the number of 45S rDNA loci (Mandáková *et al.*, 2010*a*). Thus, while chromosome number may remain stable in some closely related polyploid lineages, other chromosomal- or genomic-level changes may occur as part of the genome fractionation process.

### Concerted evolution of rDNA and gene fractionation

Although polyploidy is touted as being a process that promotes diversification, another line of thought is that the post-polyploidization diploidization processes are responsible for the great diversity generated from these whole genome duplication events (Mandáková and Lysak, 2018). The myriad of responses to the merger (through hybridization) and duplication of differentiated genomes can be lineage-specific, but most genomes ultimately respond by gene fractionation (Wendel *et al.*, 2018). As described above, the lack of expected 5S and 18S rDNA signals in the species under study here indicate that loss of these loci has occurred since polyploid formation. Our further gene sequencing of 18S rDNA indicated no sequence polymorphisms (Supplementary Data), indicating that these loci have undergone concerted evolution. The ITS sequence data (5.8S rDNA) similarly demonstrated no polymorphisms in the coding region, although we did detect some polymorphisms in the non-coding spacer regions.

Single *GBSSI-1* copies were recovered from those *Lawrencia* species included in a well-supported clade in the *GBSSI* phylogeny (Fig. 5) and the outgroups *Lecanophora chubutensis* and *R. hermaphrodita* (Table 2; Fig. 5). Chromosome counts are not available for these *Lawrencia* species, but given the *GBSSI-1* sequencing results, we would expect that these taxa would be diploid. Alternatively, if they are polyploid, then significant gene fractionation has occurred within this group, resulting in only a single copy being retained (*GBSSI-1C*). Further work on this genus would be warranted to determine chromosome number. Similarly, no chromosome count is available for *Lecanophora chubutensis*, but other species in the genus have been counted as *n* = 6, 12 or 18 (Rice *et al.*, 2015). Given that *R. hermaphrodita* is *n* = 14, this species may have also undergone gene fractionation as only a single *GBSSI-1* copy was recovered. Of particular interest, the three copies of *GBSSI* recovered from *Lawrencia berthae* corroborate an unpublished chromosome count of *n* = 21 (D. Bates, unpubl. data) for the species. Therefore, *Lawrencia berthae* is hexaploid. There is no known chromosome count for *S. hookeriana*, also from Australia, but it is likely to be a polyploid of some level based on the two copies of *GBSSI-1* (*GBSSI-1B*, *GBSSI-1C*) found here.

The *GBSSI-1* sequencing data further demonstrated differential fractionation of duplicated genes (Table 2; Fig. 5) in the Australasian lineage. Three of the *Hoheria* species maintained three *GBSSI-1* copies (*H. angustifolia*, *H. equitum* and *H. ovata*), while *H. populnea* and *H. sexstylosa* both had two copies. The two sampled individuals of *H*. *populnea* differed in their retained copies: the cultivated sample (M303) had *GBSSI-1A* and *GBSSI-1C*, while the collection from a natural population (M196) had *GBSSI-1A* and *GBSSI-1B* with *GBSSI*-*1A* seemingly a recombinant copy. We were not able to include the other two *Hoheria* species (*H. lyallii* and *H. glabrata*), but it would be interesting to determine their *GBSSI-1* profiles and compare them to the rest of the genus. Similarly, individuals of *A. discolor* showed differential loss. Including additional individuals of each species to determine the level of variability of gene fractionation at the species level would be of interest. In *Plagianthus* species, one and two copies were recovered in *P. regius* and *P. divaricatus*, respectively. As noted for the rDNA FISH results, these two species appear to be on independent trajectories with regard to their genomic constitution. Both species have lost the *GBSSI-1C* copy, as have the closely related Australian genera *Gynatrix* and *Asterotrichion*. Like *Plagianthus*, these two maintained the *GBSSI-1A* and *GBSSI-1B* copies, except that one *Asterotrichion* sample (M206) only had *GBSSI-1A.* A single loss of the *GBSSI-1C* copy in the lineage leading to *Hoheria* + *Plagianthus*, *Asterotrichion* and *Gynatrix* would be the most parsimonious explanation rather than independent loss across each individual lineage.

The recombinant *GBSSI-1A* copies identified in *H. sexstylosa* and *P. regius* were excluded from further analysis, but it is likely that non-recombinant copies are present in these species. The most probable explanation for their occurrence is an artefact of PCR, as incompletely extended DNA fragments can prime amplification from homologous, yet non-identical sequences (in this case, paralogues of *GBSSI-1*) (Bradley and Hillis, 1997). In this way, chimeric sequences are formed. Alternatively, these recombinants could be pseudogenes with their ultimate fate being lost from these genomes (Lynch and Conery, 2000). Overall, we find that *GBSSI-1* provides an interesting framework to view gene fractionation and it would be worthwhile to examine similar ‘low-copy’ nuclear genes to determine the extent of fractionation across the genome.

### Origins of polyploidy in the New Zealand endemic Malvaceae

Due to gene fractionation and loss, the origins of polyploid species can often be obscured. In our study system, as with other polyploids, nuclear ribosomal loci were not informative regarding polyploid origins of the Australasian lineage due to loss of rDNA loci as well as concerted evolution (Alvarez and Wendel, 2003; Small *et al.*, 2004). The *GBSSI-1* data, however, were somewhat informative about polyploidy in this group (Figs 5 and 6). Although loss of individual *GBSSI-1* copies was observed within species, the result of three *GBSSI-1* copies in *Lawrencia berthae* and the close relationship of these gene copies with species of *Hoheria* and *Plagianthus* indicates that polyploidization probably occurred in an ancestor of this lineage. The exact progenitor(s) for these hexaploids remains unknown but would be likely to be found in an Australian taxon (that may or may not be extinct). *Asterotrichion discolor*, with a chromosome count presented here for the first time, is probably derived from the same polyploidization event. Similarly, we would expect species of *Gynatrix*, which are found primarily in south-eastern Australia, to have a similar chromosome number, but that will await future verification. The FISH data (Fig. 2; Table 2) were not informative regarding polyploid origins but nonetheless presented insight into rDNA evolution within this group.

Polyploidy is a common feature of the New Zealand flora as well as many other island systems (Rice *et al.*, 2019; Meudt *et al.*, 2021). As many studies have employed rDNA ITS sequences for understanding phylogenetic relationships in groups that include polyploids and have not reported multiple sequence types (e.g. Barrier *et al.*, 1999; Meudt and Simpson, 2006; Mitchell *et al.*, 2009; Cantley *et al.*, 2016), we expect that concerted evolution will be a broad feature of island-dwelling polyploids. Low-copy nuclear genes can offer insight into polyploid origins (Fig. 1C; e.g. Barrier *et al.*, 1999; Harbaugh, 2008), but as the ages of the polyploids differ, identifying the parental lineages may remain challenging especially as gene fractionation occurs (Fig. 1D). A shift now toward phylogenomics of polyploids (Johnson *et al.*, 2018; Rothfels, 2020; Baker *et al.*, 2021) should be helpful in determining the level of gene fractionation and loss for other loci that follow polyploidization, which is often indicated more broadly by genome downsizing (Meudt *et al.*, 2015).

## Conclusions

Polyploids are ubiquitous in worldwide floras and are especially important components of island taxa. Although many lineages occurring on islands may show chromosomal stasis, they may also undergo further smaller-scale post-polyploidization processes of gene fractionation that ultimately lead to diploidization. Our study is one such example where the island-dwelling taxa are derived from an historical polyploidization event in the source area and further genome changes occurred post-polyploid formation in the island lineages. With new phylogenomic approaches able to sample hundreds of nuclear loci from across the genome simultaneously, we expect to gain further insight into the dynamics of polyploid genomes on islands.

## SUPPLEMENTARY DATA

Supplementary data are available online at https://academic.oup.com/aob and consist of the following: 18S rDNA alignment and voucher table with GenBank accession numbers for sequences included in this study.

mcac051_suppl_Supplementary_Data

mcac051_suppl_Supplementary_Table_S1
