# Supplementary material for: Polyploidy on islands – concerted evolution and gene loss amid chromosomal stasis
Source: Ann Bot. 2022 Apr 7;131(1):33–44. doi: 10.1093/aob/mcac051 (PMC9904340; doi:10.1093/aob/mcac051)
Supplement: mcac051_suppl_Supplementary_Data [file mcac051_suppl_supplementary_data.docx]

**Supplementary Data 2.** 18S alignment of Malvaceae included in this study. Polymorphisms are highlighted in red and with an asterisk.

1 10 20 30 40 50 60

| | | | | | |

M196_Hoheria_populnea CAACAAACCCCGACTTCTGGAAGGGATGCATTTATTAGATAAAAGGTCGACGCGGGCTTT

M198_Hoheria_sextylosa CAACAAACCCCGACTTCTGGAAGGGATGCATTTATTAGATAAAAGGTCGACGCGGGCTTT

M199_Hoheria_angustifolia CAACAAACCCCGACTTCTGGAAGGGATGCATTTATTAGATAAAAGGTCGACGCGGGCTTT

M203_Hoheria_ovata CAACAAACCCCGACTTCTGGAAGGGATGCATTTATTAGATAAAAGGTCGACGCGGGCTTT

M207_Plagianthus_regius CAACAAACCCCGACTTCTGGAAGGGATGCATTTATTAGATAAAAGGTCGACGCGGGCTTT

M197_Plag_divaricatus CAACAAACCCCGACTTCTGGAAGGGATGCATTTATTAGATAAAAGGTCGACGCGGGCTTT

M206_Asterotrichion_discolor CAACAAACCCCGACTTCTGGAAGGGATGCATTTATTAGATAAAAGGTCGACGCGGGCTTT

M211_Asterotrichion_discolor CAACAAACCCCGACTTCTGGAAGGGATGCATTTATTAGATAAAAGGTCGACGCGGGCTTT

M212_Gynatrix_pulchella CAACAAACCCCGACTTCTGGAAGGGATGCATTTATTAGATAAAAGGTCGACGCGGGCTTT

M30_L_chubutensis CAACAAACCCCGACTTCTGGAAGGGATGCATTTATTAGATAAAAGGTCGACGCGGGCTTT

M168_Lawr_squamata CAACAAACCCCGACTTCTGGAAGGGATGCATTTATTAGATAAAAGGTCGACGCGGGCTTT

M169_Lawr_virido-grisea CAACAAACCCCGACTTCTGGAAGGGATGCATTTATTAGATAAAAGGTCGACGCGGGCTTT

M172_Lawr_glomerata CAACAAACCCCGACTTCTGGAAGGGATGCATTTATTAGATAAAAGGTCGACGCGGGCTTC

M179_Lawr_glomerata CAACAAACCCCAACTTCTGGAAGGGATGCATTTATTAGATAAAAGGTCGACGCGGGCTTC

M182_Lawr_helmsii CAACAAACCCCGACTTCTGGAAGGGATGCATTTATTAGATAAAAGGTCGACGCGGGCTTT

M176_Lawr_berthae CAACAAACCCCGACTTCTGGAAGGGATGCATTTATTAGATAAAAGGTCGACGCGGGCTTT

M183_Sida_hookeriana CAACAAACCCCGACTTCTGGAAGGGATGCATTTATTAGATAAAAGGTCGACGCGGGCTTT

M187_Lawr_diffusa CAACAAACCCCGACTTCTGGAAGGGATGCATTTATTAGATAAAAGGTCGACGCGGGCTTT

M215_Ripa_hermaphrodita CAACAAACCCCGACTTCTGGAAGGGATGCATTTATTAGATAAAAGGTCGACGCGGGCTTT

M202_Hoheria_equitum CAACAAACCCCGACTTCTGGAAGGGATGCATTTATTAGATAAAAGGTCGACGCGGGCTTT

M303_Hoheria_populnea CAACAAACCCCGACTTCTGGAAGGGATGCATTTATTAGATAAAAGGTCGACGCGGGCTTT

*

61 70 80 90 100 110 120

| | | | | | |

M196_Hoheria_populnea GCCCGTTGCTCTGATGATTCATGATAACTCGACGGATCGCACGGCCTTTGTGCCGGCGAC

M198_Hoheria_sextylosa GCCCGTTGCTCTGATGATTCATGATAACTCGACGGATCGCACGGCCTTTGTGCCGGCGAC

M199_Hoheria_angustifolia GCCCGTTGCTCTGATGATTCATGATAACTCGACGGATCGCACGGCCTTTGTGCCGGCGAC

M203_Hoheria_ovata GCCCGTTGCTCTGATGATTCATGATAACTCGACGGATCGCACGGCCTTTGTGCCGGCGAC

M207_Plagianthus_regius GCCCGTTGCTCTGATGATTCATGATAACTCGACGGATCGCACGGCCTTTGTGCCGGCGAC

M197_Plag_divaricatus GCCCGTTGCTCTGATGATTCATGATAACTCGACGGATCGCACGGCCTTTGTGCCGGCGAC

M206_Asterotrichion_discolor GCCCGTTGCTCTGATGATTCATGATAACTCGACGGATCGCACGGCCTTTGTGCCGGCGAC

M211_Asterotrichion_discolor GCCCGTTGCTCTGATGATTCATGATAACTCGACGGATCGCACGGCCTTTGTGCCGGCGAC

M212_Gynatrix_pulchella GCCCGTTGCTCTGATGATTCATGATAACTCGACGGATCGCACGGCCTTTGTGCCGGCGAC

M30_L_chubutensis GCCCGTTGCTCTGATGATTCATGATAACTCGACGGATCGCACGGCCTTTGTGCCGGCGAC

M168_Lawr_squamata GCCCGTTGCCCTGATGATTCATGATAACTCGACGGATCGCACGGCCTTTGTGCCGGCGAC

M169_Lawr_virido-grisea GCCCGTTGCCCTGATGATTCATGATAACTCGACGGATCGCACGGCCTTTGTGCCGGCGAC

M172_Lawr_glomerata GCCCGTTGCCCTGATGATTCATGATAACTCGACGGATCGCACGGCCTTTGTGCCGGCGAC

M179_Lawr_glomerata GCCCGTTGCCCTGATGATTCATGATAACTCGACGGATCGCACGGCCTTTGTGCCGGCGAC

M182_Lawr_helmsii GCCCGTTGCCCTGATGATTCATGATAACTCGACGGATCGCACGGCCTTTGTGCCGGCGAC

M176_Lawr_berthae GCCCGTTGCTCTGATGATTCATGATAACTCGACGGATCGCACGGCCTTTGTGCCGGCGAC

M183_Sida_hookeriana GCCCGTTGCTCTGATGATTCATGATAACTCGACGGATCGCACGGCCTTTGTGCCGGCGAC

M187_Lawr_diffusa GCCCGTTGCTCTGATGATTCATGATAACTCGACGGATCGCACGGCCTTTGTGCCGGCGAC

M215_Ripa_hermaphrodita GCCCGTTGCTCTGATGATTCATGATAACTCGACGGATCGCACGGCCTTTGTGCCGGCGAC

M202_Hoheria_equitum GCCCGTTGCTCTGATGATTCATGATAACTCGACGGATCGCACGGCCTTTGTGCCGGCGAC

M303_Hoheria_populnea GCCCGTTGCTCTGATGATTCATGATAACTCGACGGATCGCACGGCCTTTGTGCCGGCGAC

*

121 130 140 150 160 170 180

| | | | | | |

M196_Hoheria_populnea GCATCATTCAAATTTCTGCCCTATCAACTTTCGATGGTAGGATAGTGGCCTACTATGGTG

M198_Hoheria_sextylosa GCATCATTCAAATTTCTGCCCTATCAACTTTCGATGGTAGGATAGTGGCCTACTATGGTG

M199_Hoheria_angustifolia GCATCATTCAAATTTCTGCCCTATCAACTTTCGATGGTAGGATAGTGGCCTACTATGGTG

M203_Hoheria_ovata GCATCATTCAAATTTCTGCCCTATCAACTTTCGATGGTAGGATAGTGGCCTACTATGGTG

M207_Plagianthus_regius GCATCATTCAAATTTCTGCCCTATCAACTTTCGATGGTAGGATAGTGGCCTACTATGGTG

M197_Plag_divaricatus GCATCATTCAAATTTCTGCCCTATCAACTTTCGATGGTAGGATAGTGGCCTACTATGGTG

M206_Asterotrichion_discolor GCATCATTCAAATTTCTGCCCTATCAACTTTCGATGGTAGGATAGTGGCCTACTATGGTG

M211_Asterotrichion_discolor GCATCATTCAAATTTCTGCCCTATCAACTTTCGATGGTAGGATAGTGGCCTACTATGGTG

M212_Gynatrix_pulchella GCATCATTCAAATTTCTGCCCTATCAACTTTCGATGGTAGGATAGTGGCCTACTATGGTG

M30_L_chubutensis GCATCATTCAAATTTCTGCCCTATCAACTTTCGATGGTAGGATAGTGGCCTACTATGGTG

M168_Lawr_squamata GCATCATTCAAATTTCTGCCCTATCAACTTTCGATGGTAGGATAGTGGCCTACTATGGTG

M169_Lawr_virido-grisea GCATCATTCAAATTTCTGCCCTATCAACTTTCGATGGTAGGATAGTGGCCTACTATGGTG

M172_Lawr_glomerata GCATCATTCAAATTTCTGCCCTATCAACTTTCGATGGTAGGATAGTGGCCTACTATGGTG

M179_Lawr_glomerata GCATCATTCAAATTTCTGCCCTATCAACTTTCGATGGTAGGATAGTGGCCTACTATGGTG

M182_Lawr_helmsii GCATCATTCAAATTTCTGCCCTATCAACTTTCGATGGTAGGATAGTGGCCTACTATGGTG

M176_Lawr_berthae GCATCATTCAAATTTCTGCCCTATCAACTTTCGATGGTAGGATAGTGGCCTACTATGGTG

M183_Sida_hookeriana GCATCATTCAAATTTCTGCCCTATCAACTTTCGATGGTAGGATAGTGGCCTACTATGGTG

M187_Lawr_diffusa GCATCATTCAAATTTCTGCCCTATCAACTTTCGATGGTAGGATAGTGGCCTACTATGGTG

M215_Ripa_hermaphrodita GCATCATTCAAATTTCTGCCCTATCAACTTTCGATGGTAGGATAGTGGCCTACTATGGTG

M202_Hoheria_equitum GCATCATTCAAATTTCTGCCCTATCAACTTTCGATGGTAGGATAGTGGCCTACTATGGTG

M303_Hoheria_populnea GCATCATTCAAATTTCTGCCCTATCAACTTTCGATGGTAGGATAGTGGCCTACTATGGTG

181 190 200 210 220 230 240

| | | | | | |

M196_Hoheria_populnea GTGACGGGTGACGGAGAATTAGGGTTCGATTCCGGAGAGGGAGCCTGAGAAACGGCTACC

M198_Hoheria_sextylosa GTGACGGGTGACGGAGAATTAGGGTTCGATTCCGGAGAGGGAGCCTGAGAAACGGCTACC

M199_Hoheria_angustifolia GTGACGGGTGACGGAGAATTAGGGTTCGATTCCGGAGAGGGAGCCTGAGAAACGGCTACC

M203_Hoheria_ovata GTGACGGGTGACGGAGAATTAGGGTTCGATTCCGGAGAGGGAGCCTGAGAAACGGCTACC

M207_Plagianthus_regius GTGACGGGTGACGGAGAATTAGGGTTCGATTCCGGAGAGGGAGCCTGAGAAACGGCTACC

M197_Plag_divaricatus GTGACGGGTGACGGAGAATTAGGGTTCGATTCCGGAGAGGGAGCCTGAGAAACGGCTACC

M206_Asterotrichion_discolor GTGACGGGTGACGGAGAATTAGGGTTCGATTCCGGAGAGGGAGCCTGAGAAACGGCTACC

M211_Asterotrichion_discolor GTGACGGGTGACGGAGAATTAGGGTTCGATTCCGGAGAGGGAGCCTGAGAAACGGCTACC

M212_Gynatrix_pulchella GTGACGGGTGACGGAGAATTAGGGTTCGATTCCGGAGAGGGAGCCTGAGAAACGGCTACC

M30_L_chubutensis GTGACGGGTGACGGAGAATTAGGGTTCGATTCCGGAGAGGGAGCCTGAGAAACGGCTACC

M168_Lawr_squamata GTGACGGGTGACGGAGAATTAGGGTTCGATTCCGGAGAGGGAGCCTGAGAAACGGCTACC

M169_Lawr_virido-grisea GTGACGGGTGACGGAGAATTAGGGTTCGATTCCGGAGAGGGAGCCTGAGAAACGGCTACC

M172_Lawr_glomerata GTGACGGGTGACGGAGAATTAGGGTTCGATTCCGGAGAGGGAGCCTGAGAAACGGCTACC

M179_Lawr_glomerata GTGACGGGTGACGGAGAATTAGGGTTCGATTCCGGAGAGGGAGCCTGAGAAACGGCTACC

M182_Lawr_helmsii GTGACGGGTGACGGAGAATTAGGGTTCGATTCCGGAGAGGGAGCCTGAGAAACGGCTACC

M176_Lawr_berthae GTGACGGGTGACGGAGAATTAGGGTTCGATTCCGGAGAGGGAGCCTGAGAAACGGCTACC

M183_Sida_hookeriana GTGACGGGTGACGGAGAATTAGGGTTCGATTCCGGAGAGGGAGCCTGAGAAACGGCTACC

M187_Lawr_diffusa GTGACGGGTGACGGAGAATTAGGGTTCGATTCCGGAGAGGGAGCCTGAGAAACGGCTACC

M215_Ripa_hermaphrodita GTGACGGGTGACGGAGAATTAGGGTTCGATTCCGGAGAGGGAGCCTGAGAAACGGCTACC

M202_Hoheria_equitum GTGACGGGTGACGGAGAATTAGGGTTCGATTCCGGAGAGGGAGCCTGAGAAACGGCTACC

M303_Hoheria_populnea GTGACGGGTGACGGAGAATTAGGGTTCGATTCCGGAGAGGGAGCCTGAGAAACGGCTACC

241 250 260 270 280 290 300

| | | | | | |

M196_Hoheria_populnea ACATCCAAGGAAGGCAGCAGGCGCGCAAATTACCCAATCCTGACACGGGGAGGTAGTGAC

M198_Hoheria_sextylosa ACATCCAAGGAAGGCAGCAGGCGCGCAAATTACCCAATCCTGACACGGGGAGGTAGTGAC

M199_Hoheria_angustifolia ACATCCAAGGAAGGCAGCAGGCGCGCAAATTACCCAATCCTGACACGGGGAGGTAGTGAC

M203_Hoheria_ovata ACATCCAAGGAAGGCAGCAGGCGCGCAAATTACCCAATCCTGACACGGGGAGGTAGTGAC

M207_Plagianthus_regius ACATCCAAGGAAGGCAGCAGGCGCGCAAATTACCCAATCCTGACACGGGGAGGTAGTGAC

M197_Plag_divaricatus ACATCCAAGGAAGGCAGCAGGCGCGCAAATTACCCAATCCTGACACGGGGAGGTAGTGAC

M206_Asterotrichion_discolor ACATCCAAGGAAGGCAGCAGGCGCGCAAATTACCCAATCCTGACACGGGGAGGTAGTGAC

M211_Asterotrichion_discolor ACATCCAAGGAAGGCAGCAGGCGCGCAAATTACCCAATCCTGACACGGGGAGGTAGTGAC

M212_Gynatrix_pulchella ACATCCAAGGAAGGCAGCAGGCGCGCAAATTACCCAATCCTGACACGGGGAGGTAGTGAC

M30_L_chubutensis ACATCCAAGGAAGGCAGCAGGCGCGCAAATTACCCAATCCTGACACGGGGAGGTAGTGAC

M168_Lawr_squamata ACATCCAAGGAAGGCAGCAGGCGCGCAAATTACCCAATCCTGACACGGGGAGGTAGTGAC

M169_Lawr_virido-grisea ACATCCAAGGAAGGCAGCAGGCGCGCAAATTACCCAATCCTGACACGGGGAGGTAGTGAC

M172_Lawr_glomerata ACATCCAAGGAAGGCAGCAGGCGCGCAAATTACCCAATCCTGACACGGGGAGGTAGTGAC

M179_Lawr_glomerata ACATCCAAGGAAGGCAGCAGGCGCGCAAATTACCCAATCCTGACACGGGGAGGTAGTGAC

M182_Lawr_helmsii ACATCCAAGGAAGGCAGCAGGCGCGCAAATTACCCAATCCTGACACGGGGAGGTAGTGAC

M176_Lawr_berthae ACATCCAAGGAAGGCAGCAGGCGCGCAAATTACCCAATCCTGACACGGGGAGGTAGTGAC

M183_Sida_hookeriana ACATCCAAGGAAGGCAGCAGGCGCGCAAATTACCCAATCCTGACACGGGGAGGTAGTGAC

M187_Lawr_diffusa ACATCCAAGGAAGGCAGCAGGCGCGCAAATTACCCAATCCTGACACGGGGAGGTAGTGAC

M215_Ripa_hermaphrodita ACATCCAAGGAAGGCAGCAGGCGCGCAAATTACCCAATCCTGACACGGGGAGGTAGTGAC

M202_Hoheria_equitum ACATCCAAGGAAGGCAGCAGGCGCGCAAATTACCCAATCCTGACACGGGGAGGTAGTGAC

M303_Hoheria_populnea ACATCCAAGGAAGGCAGCAGGCGCGCAAATTACCCAATCCTGACACGGGGAGGTAGTGAC

301 310 320 330 340 350 360

| | | | | | |

M196_Hoheria_populnea AATAAATAACAATACCGGGCTCAATGAGTCTGGTAATTGGAATGAGTACAATCTAAATCC

M198_Hoheria_sextylosa AATAAATAACAATACCGGGCTCAATGAGTCTGGTAATTGGAATGAGTACAATCTAAATCC

M199_Hoheria_angustifolia AATAAATAACAATACCGGGCTCAATGAGTCTGGTAATTGGAATGAGTACAATCTAAATCC

M203_Hoheria_ovata AATAAATAACAATACCGGGCTCAATGAGTCTGGTAATTGGAATGAGTACAATCTAAATCC

M207_Plagianthus_regius AATAAATAACAATACCGGGCTCAATGAGTCTGGTAATTGGAATGAGTACAATCTAAATCC

M197_Plag_divaricatus AATAAATAACAATACCGGGCTCAATGAGTCTGGTAATTGGAATGAGTACAATCTAAATCC

M206_Asterotrichion_discolor AATAAATAACAATACCGGGCTCAATGAGTCTGGTAATTGGAATGAGTACAATCTAAATCC

M211_Asterotrichion_discolor AATAAATAACAATACCGGGCTCAATGAGTCTGGTAATTGGAATGAGTACAATCTAAATCC

M212_Gynatrix_pulchella AATAAATAACAATACCGGGCTCAATGAGTCTGGTAATTGGAATGAGTACAATCTAAATCC

M30_L_chubutensis AATAAATAACAATACCGGGCTCAATGAGTCTGGTAATTGGAATGAGTACAATCTAAATCC

M168_Lawr_squamata AATAAATAACAATACCGGGCTCAATGAGTCTGGTAATTGGAATGAGTACAATCTAAATCC

M169_Lawr_virido-grisea AATAAATAACAATACCGGGCTCAATGAGTCTGGTAATTGGAATGAGTACAATCTAAATCC

M172_Lawr_glomerata AATAAATAACAATACCGGGCTCAATGAGTCTGGTAATTGGAATGAGTACAATCTAAATCC

M179_Lawr_glomerata AATAAATAACAATACCGGGCTCAATGAGTCTGGTAATTGGAATGAGTACAATCTAAATCC

M182_Lawr_helmsii AATAAATAACAATACCGGGCTCAATGAGTCTGGTAATTGGAATGAGTACAATCTAAATCC

M176_Lawr_berthae AATAAATAACAATACCGGGCTCAATGAGTCTGGTAATTGGAATGAGTACAATCTAAATCC

M183_Sida_hookeriana AATAAATAACAATACCGGGCTCAATGAGTCTGGTAATTGGAATGAGTACAATCTAAATCC

M187_Lawr_diffusa AATAAATAACAATACCGGGCTCAATGAGTCTGGTAATTGGAATGAGTACAATCTAAATCC

M215_Ripa_hermaphrodita AATAAATAACAATACCGGGCTCAATGAGTCTGGTAATTGGAATGAGTACAATCTAAATCC

M202_Hoheria_equitum AATAAATAACAATACCGGGCTCAATGAGTCTGGTAATTGGAATGAGTACAATCTAAATCC

M303_Hoheria_populnea AATAAATAACAATACCGGGCTCAATGAGTCTGGTAATTGGAATGAGTACAATCTAAATCC

361 370 380 390 400 410 420

| | | | | | |

M196_Hoheria_populnea CTTAACGAGGATCCATTGGAGGGCAAGTCTGGTGCCAGCAGCCGCGGTAATTCCAGCTCC

M198_Hoheria_sextylosa CTTAACGAGGATCCATTGGAGGGCAAGTCTGGTGCCAGCAGCCGCGGTAATTCCAGCTCC

M199_Hoheria_angustifolia CTTAACGAGGATCCATTGGAGGGCAAGTCTGGTGCCAGCAGCCGCGGTAATTCCAGCTCC

M203_Hoheria_ovata CTTAACGAGGATCCATTGGAGGGCAAGTCTGGTGCCAGCAGCCGCGGTAATTCCAGCTCC

M207_Plagianthus_regius CTTAACGAGGATCCATTGGAGGGCAAGTCTGGTGCCAGCAGCCGCGGTAATTCCAGCTCC

M197_Plag_divaricatus CTTAACGAGGATCCATTGGAGGGCAAGTCTGGTGCCAGCAGCCGCGGTAATTCCAGCTCC

M206_Asterotrichion_discolor CTTAACGAGGATCCATTGGAGGGCAAGTCTGGTGCCAGCAGCCGCGGTAATTCCAGCTCC

M211_Asterotrichion_discolor CTTAACGAGGATCCATTGGAGGGCAAGTCTGGTGCCAGCAGCCGCGGTAATTCCAGCTCC

M212_Gynatrix_pulchella CTTAACGAGGATCCATTGGAGGGCAAGTCTGGTGCCAGCAGCCGCGGTAATTCCAGCTCC

M30_L_chubutensis CTTAACGAGGATCCATTGGAGGGCAAGTCTGGTGCCAGCAGCCGCGGTAATTCCAGCTCC

M168_Lawr_squamata CTTAACGAGGATCCATTGGAGGGCAAGTCTGGTGCCAGCAGCCGCGGTAATTCCAGCTCC

M169_Lawr_virido-grisea CTTAACGAGGATCCATTGGAGGGCAAGTCTGGTGCCAGCAGCCGCGGTAATTCCAGCTCC

M172_Lawr_glomerata CTTAACGAGGATCCATTGGAGGGCAAGTCTGGTGCCAGCAGCCGCGGTAATTCCAGCTCC

M179_Lawr_glomerata CTTAACGAGGATCCATTGGAGGGCAAGTCTGGTGCCAGCAGCCGCGGTAATTCCAGCTCC

M182_Lawr_helmsii CTTAACGAGGATCCATTGGAGGGCAAGTCTGGTGCCAGCAGCCGCGGTAATTCCAGCTCC

M176_Lawr_berthae CTTAACGAGGATCCATTGGAGGGCAAGTCTGGTGCCAGCAGCCGCGGTAATTCCAGCTCC

M183_Sida_hookeriana CTTAACGAGGATCCATTGGAGGGCAAGTCTGGTGCCAGCAGCCGCGGTAATTCCAGCTCC

M187_Lawr_diffusa CTTAACGAGGATCCATTGGAGGGCAAGTCTGGTGCCAGCAGCCGCGGTAATTCCAGCTCC

M215_Ripa_hermaphrodita CTTAACGAGGATCCATTGGAGGGCAAGTCTGGTGCCAGCAGCCGCGGTAATTCCAGCTCC

M202_Hoheria_equitum CTTAACGAGGATCCATTGGAGGGCAAGTCTGGTGCCAGCAGCCGCGGTAATTCCAGCTCC

M303_Hoheria_populnea CTTAACGAGGATCCATTGGAGGGCAAGTCTGGTGCCAGCAGCCGCGGTAATTCCAGCTCC

421 430 440 450 460 470 480

| | | | | | |

M196_Hoheria_populnea AATAGCGTATATTTAAGTTGTTGCAGTTAAAAAGCTCGTAGTTGGACCTAGGGGTGGGTC

M198_Hoheria_sextylosa AATAGCGTATATTTAAGTTGTTGCAGTTAAAAAGCTCGTAGTTGGACCTAGGGGTGGGTC

M199_Hoheria_angustifolia AATAGCGTATATTTAAGTTGTTGCAGTTAAAAAGCTCGTAGTTGGACCTAGGGGTGGGTC

M203_Hoheria_ovata AATAGCGTATATTTAAGTTGTTGCAGTTAAAAAGCTCGTAGTTGGACCTAGGGGTGGGTC

M207_Plagianthus_regius AATAGCGTATATTTAAGTTGTTGCAGTTAAAAAGCTCGTAGTTGGACCTAGGGGTGGGTC

M197_Plag_divaricatus AATAGCGTATATTTAAGTTGTTGCAGTTAAAAAGCTCGTAGTTGGACCTAGGGGTGGGTC

M206_Asterotrichion_discolor AATAGCGTATATTTAAGTTGTTGCAGTTAAAAAGCTCGTAGTTGGACCTAGGGGTGGGTC

M211_Asterotrichion_discolor AATAGCGTATATTTAAGTTGTTGCAGTTAAAAAGCTCGTAGTTGGACCTAGGGGTGGGTC

M212_Gynatrix_pulchella AATAGCGTATATTTAAGTTGTTGCAGTTAAAAAGCTCGTAGTTGGACCTAGGGGTGGGTC

M30_L_chubutensis AATAGCGTATATTTAAGTTGTTGCAGTTAAAAAGCTCGTAGTTGGACTTAGGGTTGGGTT

M168_Lawr_squamata AATAGCGTATATTTAAGTTGTTGCAGTTAAAAAGCTCGTAGTTGGACCTAGGGGTGGGTC

M169_Lawr_virido-grisea AATAGCGTATATTTAAGTTGTTGCAGTTAAAAAGCTCGTAGTTGGACCTAGGGGTGGGTC

M172_Lawr_glomerata AATAGCGTATATTTAAGTTGTTGCAGTTAAAAAGCTCGTAGTTGGACCTAGGGGTGGGTC

M179_Lawr_glomerata AATAGCGTATATTTAAGTTGTTGCAGTTAAAAAGCTCGTAGTTGGACCTAGGGGTGGGTC

M182_Lawr_helmsii AATAGCGTATATTTAAGTTGTTGCAGTTAAAAAGCTCGTAGTTGAACCTAGGGGTGGGTC

M176_Lawr_berthae AATAGCGTATATTTAAGTTGTTGCAGTTAAAAAGCTCGTAGTTGGACCTAGGGGTGTGTC

M183_Sida_hookeriana AATAGCGTATATTTAAGTTGTTGCAGTTAAAAAGCTCGTAGTTGGACCTAGGGGTGGGTC

M187_Lawr_diffusa AATAGCGTATATTTAAGTTGTTGCAGTTAAAAAGCTCGTAGTTGGACCTAGGGGTGGGTC

M215_Ripa_hermaphrodita AATAGCGTATATTTAAGTTGTTGCAGTTAAAAAGCTCGTAGTTGGACCTAGGGGTGGGTC

M202_Hoheria_equitum AATAGCGTATATTTAAGTTGTTGCAGTTAAAAAGCTCGTAGTTGGACCTAGGGGTGGGTC

M303_Hoheria_populnea AATAGCGTATATTTAAGTTGTTGCAGTTAAAAAGCTCGTAGTTGGACCTAGGGGTGGGTC

* * *

481 490 500 510 520 530 540

| | | | | | |

M196_Hoheria_populnea TTCCGGTCCGCCTCACGGTGAGCACCGGTCGGCTCGTCCCTACTGCCGGCGATGCGCTCC

M198_Hoheria_sextylosa TTCCGGTCCGCCTCACGGTGAGCACCGGTCGGCTCGTCCCTACTGCCGGCGATGCGCTCC

M199_Hoheria_angustifolia TTCCGGTCCGCCTCACGGTGAGCACCGGTCGGCTCGTCCCTACTGCCGGCGATGCGCTCC

M203_Hoheria_ovata TTCCGGTCCGCCTCACGGTGAGCACCGGTCGGCTCGTCCCTACTGCCGGCGATGCGCTCC

M207_Plagianthus_regius TTCCGGTCCGCCTCACGGTGAGCACCGGTCGGCTCGTCCCTACTGCCGGCGATGCGCTCC

M197_Plag_divaricatus TTCCGGTCCGCCTCGCGGTGAGCACCGGTCGGCTCGTCCCTACTGCCGGCGATGCGCTCC

M206_Asterotrichion_discolor TTCCGGTCCGCCTCACGGTGAGCACCGGTCGGCTCGTCCCTACTGCCGGCGATGCGCTCC

M211_Asterotrichion_discolor TTCCGGTCCGCCTCACGGTGAGCACCGGTCGGCTCGTCCCTACTGCCGGCGATGCGCTCC

M212_Gynatrix_pulchella TTCCGGTCCGCCTCACGGTGAGCACCGGTCGGCTCGTCCCTACTGCCGGCGATGCGCTCC

M30_L_chubutensis GTCAGGTCCGCCTTACGGTGTGCACCGAATAGCTCGTCCCTACTGCCGGCGATGCGCTCC

M168_Lawr_squamata TTCCGGTCCGCCTCACGGTGAGCACCGGTCGGCTCGTCCCTTCTGCCGGCGATGCGCTCC

M169_Lawr_virido-grisea TTCCGGTCCGCCTCACGGTGAGCACCGGTCGGCTCGTCCCTTCTGCCGGCGATGCGCTCC

M172_Lawr_glomerata TTCCGGTCCGCCTCACGGTGAGCACCGGTCGGCTCGTCCCTACTGCCGGCGATGCGCTCC

M179_Lawr_glomerata TTCCGGTCCGCCTCACGGTGAGCACCGGTCGGCTCGTCCCTACTGCCGGCGATGCGCTCC

M182_Lawr_helmsii TTCCGGTCCGCCTCACGGTGAGCACCGGTCGGCTCGTCCCTTCTGCCGGCGATGCGCTCC

M176_Lawr_berthae TTCCGGTCCGCCTCACGGTGAGCACCGGTCGGCTCGTCCCTACTGCCGGCGATGCGCTCC

M183_Sida_hookeriana TTCCGGTCCGCCTCACGGTGAGCACCGGTCGGCTCGTCCCTACTGCCGGCGATGCGCTCC

M187_Lawr_diffusa TTCCGGTCCGCCTCACGGTGAGCACCGGTCGGCTCGTCCCTACTGCCGGCGATGCGCTCC

M215_Ripa_hermaphrodita TTCCGGTCCGCCTTCCGGTGAGCACCGGTCGGCTCGTCCCTACTGCCGGCGATGCGCTCC

M202_Hoheria_equitum TTCCGGTCCGCCTCACGGTGAGCACCGGTCGGCTCGTCCCTACTGCCGGCGATGCGCTCC

M303_Hoheria_populnea TTCCGGTCCGCCTCACGGTGAGCACCGGTCGGCTCGTCCCTACTGCCGGCGATGCGCTCC

* * ** * **** *

541 550 560 570 580 590 600

| | | | | | |

M196_Hoheria_populnea TGGCCTTAATTGGCCGGGTCGTTCCTCCGGCGCTGTTACTTTGAAGAAATTAGAGTGCTC

M198_Hoheria_sextylosa TGGCCTTAATTGGCCGGGTCGTTCCTCCGGCGCTGTTACTTTGAAGAAATTAGAGTGCTC

M199_Hoheria_angustifolia TGGCCTTAATTGGCCGGGTCGTTCCTCCGGCGCTGTTACTTTGAAGAAATTAGAGTGCTC

M203_Hoheria_ovata TGGCCTTAATTGGCCGGGTCGTTCCTCCGGCGCTGTTACTTTGAAGAAATTAGAGTGCTC

M207_Plagianthus_regius TGGCCTTAATTGGCCGGGTCGTTCCTCCGGCGCTGTTACTTTGAAGAAATTAGAGTGCTC

M197_Plag_divaricatus TGGCCTTAATTGGCCGGGTCGTTCCTCCGGCGCTGTTACTTTGAAGAAATTAGAGTGCTC

M206_Asterotrichion_discolor TGGCCTTAATTGGCCGGGTCGTTCCTCCGGCGCTGTTACTTTGAAGAAATTAGAGTGCTC

M211_Asterotrichion_discolor TGGCCTTAATTGGCCGGGTCGTTCCTCCGGCGCTGTTACTTTGAAGAAATTAGAGTGCTC

M212_Gynatrix_pulchella TGGCCTTAATTGGCCGGGTCGTTCCTCCGGCGCTGTTACTTTGAAGAAATTAGAGTGCTC

M30_L_chubutensis TGGCCTTAATTGGCCGGGTCGTTCCTCCGGCGCTGTTACTTTGAAGAAATTAGAGTGCTC

M168_Lawr_squamata TGGCCTTAATTGGCCGGGTCGTTCCTCCGGCGCTGTTACTTTGAAGAAATTAGAGTGCTC

M169_Lawr_virido-grisea TGGCCTTAATTGGCCGGGTCGTTCCTCCGGCGCTGTTACTTTGAAGAAATTAGAGTGCTC

M172_Lawr_glomerata TGGCCTTAATTGGCCGGGTCGTTCCTCCGGCGCTGTTACTTTGAAGAAATTAGAGTGCTC

M179_Lawr_glomerata TGGCCTTAATTGGCCGGGTCGTTCCTCCGGCGCTGTTACTTTGAAGAAATTAGAGTGCTC

M182_Lawr_helmsii TGGCCTTAATTGGCCGGGTCGTTCCTCCGGCGCTGTTACTTTGAAGAAATTAGAGTGCTC

M176_Lawr_berthae TGGCCTTAATTGGCCGGGTCGTTCCTCCGGCGCTGTTACTTTGAAGAAATTAGAGTGCTC

M183_Sida_hookeriana TGGCCTTAATTGGCCGGGTCGTTCCTCCGGCGCTGTTACTTTGAAGAAATTAGAGTGCTC

M187_Lawr_diffusa TGGCCTTAATTGGCCGGGTCGTTCCTCCGGCGCTGTTACTTTGAAGAAATTAGAGTGCTC

M215_Ripa_hermaphrodita TGGCCTTAATTGGCCGGGTCGTTCCTCCGGCGCTGTTACTTTGAAGAAATTAGAGTGCTC

M202_Hoheria_equitum TGGCCTTAATTGGCCGGGTCGTTCCTCCGGCGCTGTTACTTTGAAGAAATTAGAGTGCTC

M303_Hoheria_populnea TGGCCTTAATTGGCCGGGTCGTTCCTCCGGCGCTGTTACTTTGAAGAAATTAGAGTGCTC

601 610 620 630 640 650 660

| | | | | | |

M196_Hoheria_populnea AAAGCAGGCCTACGCTTGTATACATTAGCATGGGATAACATCATAGGATTTCGGTCCTAT

M198_Hoheria_sextylosa AAAGCAGGCCTACGCTTGTATACATTAGCATGGGATAACATCATAGGATTTCGGTCCTAT

M199_Hoheria_angustifolia AAAGCAGGCCTACGCTTGTATACATTAGCATGGGATAACATCATAGGATTTCGGTCCTAT

M203_Hoheria_ovata AAAGCAGGCCTACGCTTGTATACATTAGCATGGGATAACATCATAGGATTTCGGTCCTAT

M207_Plagianthus_regius AAAGCAGGCCTACGCTTGTATACATTAGCATGGGATAACATCATAGGATTTCGGTCCTAT

M197_Plag_divaricatus AAAGCAGGCCTACGCTTGTATACATTAGCATGGGATAACATCATAGGATTTCGGTCCTAT

M206_Asterotrichion_discolor AAAGCAGGCCTACGCTTGTATACATTAGCATGGGATAACATCATAGGATTTCGGTCCTAT

M211_Asterotrichion_discolor AAAGCAGGCCTACGCTTGTATACATTAGCATGGGATAACATCATAGGATTTCGGTCCTAT

M212_Gynatrix_pulchella AAAGCAGGCCTACGCTTGTATACATTAGCATGGGATAACATCATAGGATTTCGGTCCTAT

M30_L_chubutensis AAAGCAGGCCTACGCTTGTATACATTAGCATGGGATAACATCATAGGATTTCGATCCTAT

M168_Lawr_squamata AAAGCAGGCCTACGCTTGTATACATTAGCATGGGATAACATCATAGGATTTCGGTCCTAT

M169_Lawr_virido-grisea AAAGCAGGCCTACGCCTGTATACATTAGCATGGGATAACATCATAGGATTTCGGTCCTAT

M172_Lawr_glomerata AAAGCAGGCCTACGCTTGTATACATTAGCATGGGATAACATCATAGGATTTCGGTCCTAT

M179_Lawr_glomerata AAAGCAGGCCTACGCTTGTATACATTAGCATGGGATAACATCATAGGATTTCGGTCCTAT

M182_Lawr_helmsii AAAGCAGGCCTACGCTTGTATACATTAGCATGGGATAACATCATAGGATTTCGGTCCTAT

M176_Lawr_berthae AAAGCAGGCCTACGCTTGTATACATTAGCATGGGATAACATCATAGGATTTCGGTCCTAT

M183_Sida_hookeriana AAAGCAGGCCTACGCTTGTATACATTAGCATGGGATAACATCATAGGATTTCGGTCCTAT

M187_Lawr_diffusa AAAGCAGGCCTACGCTTGTATACATTAGCATGGGATAACATCATAGGATTTCGGTCCTAT

M215_Ripa_hermaphrodita AAAGCAGGCCTACGCTTGTATACATTAGCATGGGATAACATCATAGGATTTCGGTCCTAT

M202_Hoheria_equitum AAAGCAGGCCTACGCTTGTATACATTAGCATGGGATAACATCATAGGATTTCGGTCCTAT

M303_Hoheria_populnea AAAGCAGGCCTACGCTTGTATACATTAGCATGGGATAACATCATAGGATTTCGGTCCTAT

- *

661 670 680 690 700 710 720

| | | | | | |

M196_Hoheria_populnea TCTGTTGGCCTTCGGGATCGGAGTAATGATTAACAGGGACAGTCGGGGGCATTCGTATTT

M198_Hoheria_sextylosa TCTGTTGGCCTTCGGGATCGGAGTAATGATTAACAGGGACAGTCGGGGGCATTCGTATTT

M199_Hoheria_angustifolia TCTGTTGGCCTTCGGGATCGGAGTAATGATTAACAGGGACAGTCGGGGGCATTCGTATTT

M203_Hoheria_ovata TCTGTTGGCCTTCGGGATCGGAGTAATGATTAACAGGGACAGTCGGGGGCATTCGTATTT

M207_Plagianthus_regius TCTGTTGGCCTTCGGGATCGGAGTAATGATTAACAGGGACAGTCGGGGGCATTCGTATTT

M197_Plag_divaricatus TCTGTTGGCCTTCGGGATCGGAGTAATGATTAACAGGGACAGTCGGGGGCATTCGTATTT

M206_Asterotrichion_discolor TCTGTTGGCCTTCGGGATCGGAGTAATGATTAACAGGGACAGTCGGGGGCATTCGTATTT

M211_Asterotrichion_discolor TCTGTTGGCCTTCGGGATCGGAGTAATGATTAACAGGGACAGTCGGGGGCATTCGTATTT

M212_Gynatrix_pulchella TCTGTTGGCCTTCGGGATCGGAGTAATGATTAACAGGGACAGTCGGGGGCATTCGTATTT

M30_L_chubutensis TCTGTTGGCCTTCGGGATCGGAGTAATGATTAACAGGGACAGTCGGGGGCATTCGTATTT

M168_Lawr_squamata TCTGTTGGCCTTCGGGATCGGAGTAATGATTAACAGGGACAGTCGGGGGCATTCGTATTT

M169_Lawr_virido-grisea TCTGTTGGCCTTCGGGATCGGAGTAATGATTAACAGGGACAGTCGGGGGCATTCGTATTT

M172_Lawr_glomerata TCTGTTGGCCTTCGGGATCGGAGTAATGATTAACAGGGACAGTCGGGGGCATTCGTATTT

M179_Lawr_glomerata TCTGTTGGCCTTCGGGATCGGAGTAATGATTAACAGGGACAGTCGGGGGCATTCGTATTT

M182_Lawr_helmsii TCTGTTGGCCTTCGGGATCGGAGTAATGATTAACAGGGACAGTCGGGGGCATTCGTATTT

M176_Lawr_berthae TCTGTTGGCCTTCGGGATCGGAGTAATGATTAACAGGGACAGTCGGGGGCATTCGTATTT

M183_Sida_hookeriana TCTGTTGGCCTTCGGGATCGGAGTAATGATTAACAGGGACAGTCGGGGGCATTCGTATTT

M187_Lawr_diffusa TCTGTTGGCCTTCGGGATCGGAGTAATGATTAACAGGGACAGTCGGGGGCATTCGTATTT

M215_Ripa_hermaphrodita TCTGTTGGCCTTCGGGATCGGAGTAATGATTAACAGGGACAGTCGGGGGCATTCGTATTT

M202_Hoheria_equitum TCTGTTGGCCTTCGGGATCGGAGTAATGATTAACAGGGACAGTCGGGGGCATTCGTATTT

M303_Hoheria_populnea TCTGTTGGCCTTCGGGATCGGAGTAATGATTAACAGGGACAGTCGGGGGCATTCGTATTT

721 730 740 750 760 770 780

| | | | | | |

M196_Hoheria_populnea CATAGTCAGAGGTGAAATTCTTGGATTTATGAAAGACGAACAACTGCGAAAGCATTTGCC

M198_Hoheria_sextylosa CATAGTCAGAGGTGAAATTCTTGGATTTATGAAAGACGAACAACTGCGAAAGCATTTGCC

M199_Hoheria_angustifolia CATAGTCAGAGGTGAAATTCTTGGATTTATGAAAGACGAACAACTGCGAAAGCATTTGCC

M203_Hoheria_ovata CATAGTCAGAGGTGAAATTCTTGGATTTATGAAAGACGAACAACTGCGAAAGCATTTGCC

M207_Plagianthus_regius CATAGTCAGAGGTGAAATTCTTGGATTTATGAAAGACGAACAACTGCGAAAGCATTTGCC

M197_Plag_divaricatus CATAGTCAGAGGTGAAATTCTTGGATTTATGAAAGACGAACAACTGCGAAAGCATTTGCC

M206_Asterotrichion_discolor CATAGTCAGAGGTGAAATTCTTGGATTTATGAAAGACGAACAACTGCGAAAGCATTTGCC

M211_Asterotrichion_discolor CATAGTCAGAGGTGAAATTCTTGGATTTATGAAAGACGAACAACTGCGAAAGCATTTGCC

M212_Gynatrix_pulchella CATAGTCAGAGGTGAAATTCTTGGATTTATGAAAGACGAACAACTGCGAAAGCATTTGCC

M30_L_chubutensis CATAGTCAGAGGTGAAATTCTTGGATTTATGAAAGACGAACAACTGCGAAAGCATTTGCC

M168_Lawr_squamata CATAGTCAGAGGTGAAATTCTTGGATTTATGAAAGACGAACAACTGCGAAAGCATTTGCC

M169_Lawr_virido-grisea CATAGTCAGAGGTGAAATTCTTGGATTTATGAAAGACGAACAACTGCGAAAGCATTTGCC

M172_Lawr_glomerata CATAGTCAGAGGTGAAATTCTTGGATTTATGAAAGACGAACAACTGCGAAAGCATTTGCC

M179_Lawr_glomerata CATAGTCAGAGGTGAAATTCTTGGATTTATGAAAGACGAACAACTGCGAAAGCATTTGCC

M182_Lawr_helmsii CATAGTCAGAGGTGAAATTCTTGGATTTATGAAAGACGAACAACTGCGAAAGCATTTGCC

M176_Lawr_berthae CATAGTCAGAGGTGAAATTCTTGGATTTATGAAAGACGAACAACTGCGAAAGCATTTGCC

M183_Sida_hookeriana CATAGTCAGAGGTGAAATTCTTGGATTTATGAAAGACGAACAACTGCGAAAGCATTTGCC

M187_Lawr_diffusa CATAGTCAGAGGTGAAATTCTTGGATTTATGAAAGACGAACAACTGCGAAAGCATTTGCC

M215_Ripa_hermaphrodita CATAGTCAGAGGTGAAATTCTTGGATTTATGAAAGACGAACAACTGCGAAAGCATTTGCC

M202_Hoheria_equitum CATAGTCAGAGGTGAAATTCTTGGATTTATGAAAGACGAACAACTGCGAAAGCATTTGCC

M303_Hoheria_populnea CATAGTCAGAGGTGAAATTCTTGGATTTATGAAAGACGAACAACTGCGAAAGCATTTGCC

781 790 800 810 820 830 840

| | | | | | |

M196_Hoheria_populnea AAGGATGTTTTCATTAATCAAGAACGAAAGTTGGGGGCTCGAAGACGATCAGATACCGTC

M198_Hoheria_sextylosa AAGGATGTTTTCATTAATCAAGAACGAAAGTTGGGGGCTCGAAGACGATCAGATACCGTC

M199_Hoheria_angustifolia AAGGATGTTTTCATTAATCAAGAACGAAAGTTGGGGGCTCGAAGACGATCAGATACCGTC

M203_Hoheria_ovata AAGGATGTTTTCATTAATCAAGAACGAAAGTTGGGGGCTCGAAGACGATCAGATACCGTC

M207_Plagianthus_regius AAGGATGTTTTCATTAATCAAGAACGAAAGTTGGGGGCTCGAAGACGATCAGATACCGTC

M197_Plag_divaricatus AAGGATGTTTTCATTAATCAAGAACGAAAGTTGGGGGCTCGAAGACGATCAGATACCGTC

M206_Asterotrichion_discolor AAGGATGTTTTCATTAATCAAGAACGAAAGTTGGGGGCTCGAAGACGATCAGATACCGTC

M211_Asterotrichion_discolor AAGGATGTTTTCATTAATCAAGAACGAAAGTTGGGGGCTCGAAGACGATCAGATACCGTC

M212_Gynatrix_pulchella AAGGATGTTTTCATTAATCAAGAACGAAAGTTGGGGGCTCGAAGACGATCAGATACCGTC

M30_L_chubutensis AAGGATGTTTTCATTAATCAAGAACGAAAGTTGGGGGCTCGAAGACGATCAGATACCGTC

M168_Lawr_squamata AAGGATGTTTTCATTAATCAAGAACGAAAGTTGGGGGCTCGAAGACGATCAGATACCGTC

M169_Lawr_virido-grisea AAGGATGTTTTCATTAATCAAGAACGAAAGTTGGGGGCTCGAAGACGATCAGATACCGTC

M172_Lawr_glomerata AAGGATGTTTTCATTAATCAAGAACGAAAGTTGGGGGCTCGAAGACGATCAGATACCGTC

M179_Lawr_glomerata AAGGATGTTTTCATTAATCAAGAACGAAAGTTGGGGGCTCGAAGACGATCAGATACCGTC

M182_Lawr_helmsii AAGGATGTTTTCATTAATCAAGAACGAAAGTTGGGGGCTCGAAGACGATCAGATACCGTC

M176_Lawr_berthae AAGGATGTTTTCATTAATCAAGAACGAAAGTTGGGGGCTCGAAGACGATCAGATACCGTC

M183_Sida_hookeriana AAGGATGTTTTCATTAATCAAGAACGAAAGTTGGGGGCTCGAAGACGATCAGATACCGTC

M187_Lawr_diffusa AAGGATGTTTTCATTAATCAAGAACGAAAGTTGGGGGCTCGAAGACGATCAGATACCGTC

M215_Ripa_hermaphrodita AAGGATGTTTTCATTAATCAAGAACGAAAGTTGGGGGCTCGAAGACGATCAGATACCGTC

M202_Hoheria_equitum AAGGATGTTTTCATTAATCAAGAACGAAAGTTGGGGGCTCGAAGACGATCAGATACCGTC

M303_Hoheria_populnea AAGGATGTTTTCATTAATCAAGAACGAAAGTTGGGGGCTCGAAGACGATCAGATACCGTC

841 850 860 870 880 890 900

| | | | | | |

M196_Hoheria_populnea CTAGTCTCAACCATAAACGATGCCGACCAGGGATCGGCGGATGTTGCTTTTAGGACTCCG

M198_Hoheria_sextylosa CTAGTCTCAACCATAAACGATGCCGACCAGGGATCGGCGGATGTTGCTTTTAGGACTCCG

M199_Hoheria_angustifolia CTAGTCTCAACCATAAACGATGCCGACCAGGGATCGGCGGATGTTGCTTTTAGGACTCCG

M203_Hoheria_ovata CTAGTCTCAACCATAAACGATGCCGACCAGGGATCGGCGGATGTTGCTTTTAGGACTCCG

M207_Plagianthus_regius CTAGTCTCAACCATAAACGATGCCGACCAGGGATCGGCGGATGTTGCTTTTAGGACTCCG

M197_Plag_divaricatus CTAGTCTCAACCATAAACGATGCCGACCAGGGATCGGCGGATGTTGCTTTTAGGACTCCG

M206_Asterotrichion_discolor CTAGTCTCAACCATAAACGATGCCGACCAGGGATCGGCGGATGTTGCTTTTAGGACTCCG

M211_Asterotrichion_discolor CTAGTCTCAACCATAAACGATGCCGACCAGGGATCGGCGGATGTTGCTTTTAGGACTCCG

M212_Gynatrix_pulchella CTAGTCTCAACCATAAACGATGCCGACCAGGGATCGGCGGATGTTGCTTTTAGGACTCCG

M30_L_chubutensis CTAGTCTCAACCATAAACGATGCCGACCAGGGATCGGCGGATGTTGCTTTTAGGACTCCG

M168_Lawr_squamata CTAGTCTCAACCATAAACGATGCCGACCAGGGATCGGCGGATGTTGCTTTTAGGACTCCG

M169_Lawr_virido-grisea CTAGTCTCAACCATAAACGATGCCGACCAGGGATCGGCGGATGTTGCTTTTAGGACTCCG

M172_Lawr_glomerata CTAGTCTCAACCATAAACGATGCCGACCAGGGATCGGCGGATGTTGCTTTTAGGACTCCG

M179_Lawr_glomerata CTAGTCTCAACCATAAACGATGCCGACCAGGGATCGGCGGATGTTGCTTTTAGGACTCCG

M182_Lawr_helmsii CTAGTCTCAACCATAAACGATGCCGACCAGGGATCGGCGGATGTTGCTTTTAGGACTCCG

M176_Lawr_berthae CTAGTCTCAACCATAAACGATGCCGACCAGGGATCGGCGGATGTTGCTTTTAGGACTCCG

M183_Sida_hookeriana CTAGTCTCAACCATAAACGATGCCGACCAGGGATCGGCGGATGTTGCTTTTAGGACTCCG

M187_Lawr_diffusa CTAGTCTCAACCATAAACGATGCCGACCAGGGATCGGCGGATGTTGCTTTTAGGACTCCG

M215_Ripa_hermaphrodita CTAGTCTCAACCATAAACGATGCCGACCAGGGATCGGCGGATGTTGCTTTTAGGACTCCG

M202_Hoheria_equitum CTAGTCTCAACCATAAACGATGCCGACCAGGGATCGGCGGATGTTGCTTTTAGGACTCCG

M303_Hoheria_populnea CTAGTCTCAACCATAAACGATGCCGACCAGGGATCGGCGGATGTTGCTTTTAGGACTCCG

901 910 920 930 940 950 960

| | | | | | |

M196_Hoheria_populnea CCGGCACCTTATGAGAAATCAAAGTCTTTGGGTTCCGGGGGGAGTATGGTCGCAAGGCTG

M198_Hoheria_sextylosa CCGGCACCTTATGAGAAATCAAAGTCTTTGGGTTCCGGGGGGAGTATGGTCGCAAGGCTG

M199_Hoheria_angustifolia CCGGCACCTTATGAGAAATCAAAGTCTTTGGGTTCCGGGGGGAGTATGGTCGCAAGGCTG

M203_Hoheria_ovata CCGGCACCTTATGAGAAATCAAAGTCTTTGGGTTCCGGGGGGAGTATGGTCGCAAGGCTG

M207_Plagianthus_regius CCGGCACCTTATGAGAAATCAAAGTCTTTGGGTTCCGGGGGGAGTATGGTCGCAAGGCTG

M197_Plag_divaricatus CCGGCACCTTATGAGAAATCAAAGTCTTTGGGTTCCGGGGGGAGTATGGTCGCAAGGCTG

M206_Asterotrichion_discolor CCGGCACCTTATGAGAAATCAAAGTCTTTGGGTTCCGGGGGGAGTATGGTCGCAAGGCTG

M211_Asterotrichion_discolor CCGGCACCTTATGAGAAATCAAAGTCTTTGGGTTCCGGGGGGAGTATGGTCGCAAGGCTG

M212_Gynatrix_pulchella CCGGCACCTTATGAGAAATCAAAGTCTTTGGGTTCCGGGGGGAGTATGGTCGCAAGGCTG

M30_L_chubutensis CCGGCACCTTATGAGAAATCAAAGTCTTTGGGTTCCGGGGGGAGTATGGTCGCAAGGCTG

M168_Lawr_squamata CCGGCACCTTATGAGAAATCAAAGTCTTTGGGTTCCGGGGGGAGTATGGTCGCAAGGCTG

M169_Lawr_virido-grisea CCGGCACCTTATGAGAAATCAAAGTCTTTGGGTTCCGGGGGGAGTATGGTCGCAAGGCTG

M172_Lawr_glomerata CCGGCACCTTATGAGAAATCAAAGTCTTTGGGTTCCGGGGGGAGTATGGTCGCAAGGCTG

M179_Lawr_glomerata CCGGCACCTTATGAGAAATCAAAGTCTTTGGGTTCCGGGGGGAGTATGGTCGCAAGGCTG

M182_Lawr_helmsii CCGGCACCTTATGAGAAATCAAAGTCTTTGGGTTCCGGGGGGAGTATGGTCGCAAGGCTG

M176_Lawr_berthae CCGGCACCTTATGAGAAATCAAAGTCTTTGGGTTCCGGGGGGAGTATGGTCGCAAGGCTG

M183_Sida_hookeriana CCGGCACCTTATGAGAAATCAAAGTCTTTGGGTTCCGGGGGGAGTATGGTCGCAAGGCTG

M187_Lawr_diffusa CCGGCACCTTATGAGAAATCAAAGTCTTTGGGTTCCGGGGGGAGTATGGTCGCAAGGCTG

M215_Ripa_hermaphrodita CCGGCACCTTATGAGAAATCAAAGTCTTTGGGTTCCGGGGGGAGTATGGTCGCAAGGCTG

M202_Hoheria_equitum CCGGCACCTTATGAGAAATCAAAGTCTTTGGGTTCCGGGGGGAGTATGGTCGCAAGGCTG

M303_Hoheria_populnea CCGGCACCTTATGAGAAATCAAAGTCTTTGGGTTCCGGGGGGAGTATGGTCGCAAGGCTG

961 970 980 990 1000 1010 1020

| | | | | | |

M196_Hoheria_populnea AAACTTAAAGGAATTGACGGAAGGGCACCACCAGGAGTGGAGCCTGCGGCTTAATTTGAC

M198_Hoheria_sextylosa AAACTTAAAGGAATTGACGGAAGGGCACCACCAGGAGTGGAGCCTGCGGCTTAATTTGAC

M199_Hoheria_angustifolia AAACTTAAAGGAATTGACGGAAGGGCACCACCAGGAGTGGAGCCTGCGGCTTAATTTGAC

M203_Hoheria_ovata AAACTTAAAGGAATTGACGGAAGGGCACCACCAGGAGTGGAGCCTGCGGCTTAATTTGAC

M207_Plagianthus_regius AAACTTAAAGGAATTGACGGAAGGGCACCACCAGGAGTGGAGCCTGCGGCTTAATTTGAC

M197_Plag_divaricatus AAACTTAAAGGAATTGACGGAAGGGCACCACCAGGAGTGGAGCCTGCGGCTTAATTTGAC

M206_Asterotrichion_discolor AAACTTAAAGGAATTGACGGAAGGGCACCACCAGGAGTGGAGCCTGCGGCTTAATTTGAC

M211_Asterotrichion_discolor AAACTTAAAGGAATTGACGGAAGGGCACCACCAGGAGTGGAGCCTGCGGCTTAATTTGAC

M212_Gynatrix_pulchella AAACTTAAAGGAATTGACGGAAGGGCACCACCAGGAGTGGAGCCTGCGGCTTAATTTGAC

M30_L_chubutensis AAACTTAAAGGAATTGACGGAAGGGCACCACCAGGAGTGGAGCCTGCGGCTTAATTTGAC

M168_Lawr_squamata AAACTTAAAGGAATTGACGGAAGGGCACCACCAGGAGTGGAGCCTGCGGCTTAATTTGAC

M169_Lawr_virido-grisea AAACTTAAAGGAATTGACGGAAGGGCACCACCAGGAGTGGAGCCTGCGGCTTAATTTGAC

M172_Lawr_glomerata AAACTTAAAGGAATTGACGGAAGGGCACCACCAGGAGTGGAGCCTGCGGCTTAATTTGAC

M179_Lawr_glomerata AAACTTAAAGGAATTGACGGAAGGGCACCACCAGGAGTGGAGCCTGCGGCTTAATTTGAC

M182_Lawr_helmsii AAACTTAAAGGAATTGACGGAAGGGCACCACCAGGAGTGGAGCCTGCGGCTTAATTTGAC

M176_Lawr_berthae AAACTTAAAGGAATTGACGGAAGGGCACCACCAGGAGTGGAGCCTGCGGCTTAATTTGAC

M183_Sida_hookeriana AAACTTAAAGGAATTGACGGAAGGGCACCACCAGGAGTGGAGCCTGCGGCTTAATTTGAC

M187_Lawr_diffusa AAACTTAAAGGAATTGACGGAAGGGCACCACCAGGAGTGGAGCCTGCGGCTTAATTTGAC

M215_Ripa_hermaphrodita AAACTTAAAGGAATTGACGGAAGGGCACCACCAGGAGTGGAGCCTGCGGCTTAATTTGAC

M202_Hoheria_equitum AAACTTAAAGGAATTGACGGAAGGGCACCACCAGGAGTGGAGCCTGCGGCTTAATTTGAC

M303_Hoheria_populnea AAACTTAAAGGAATTGACGGAAGGGCACCACCAGGAGTGGAGCCTGCGGCTTAATTTGAC

1021 1030 1040 1050 1060 1070 1080

| | | | | | |

M196_Hoheria_populnea TCAACACGGGGAAACTTACCAGGTCCAGACATAGTAAGGATTGACAGACTGAGAGCTCTT

M198_Hoheria_sextylosa TCAACACGGGGAAACTTACCAGGTCCAGACATAGTAAGGATTGACAGACTGAGAGCTCTT

M199_Hoheria_angustifolia TCAACACGGGGAAACTTACCAGGTCCAGACATAGTAAGGATTGACAGACTGAGAGCTCTT

M203_Hoheria_ovata TCAACACGGGGAAACTTACCAGGTCCAGACATAGTAAGGATTGACAGACTGAGAGCTCTT

M207_Plagianthus_regius TCAACACGGGGAAACTTACCAGGTCCAGACATAGTAAGGATTGACAGACTGAGAGCTCTT

M197_Plag_divaricatus TCAACACGGGGAAACTTACCAGGTCCAGACATAGTAAGGATTGACAGACTGAGAGCTCTT

M206_Asterotrichion_discolor TCAACACGGGGAAACTTACCAGGTCCAGACATAGTAAGGATTGACAGACTGAGAGCTCTT

M211_Asterotrichion_discolor TCAACACGGGGAAACTTACCAGGTCCAGACATAGTAAGGATTGACAGACTGAGAGCTCTT

M212_Gynatrix_pulchella TCAACACGGGGAAACTTACCAGGTCCAGACATAGTAAGGATTGACAGACTGAGAGCTCTT

M30_L_chubutensis TCAACACGGGGAAACTTACCAGGTCCAGACATAGTAAGGATTGACAGACTGAGAGCTCTT

M168_Lawr_squamata TCAACACGGGGAAACTTACCAGGTCCAGACATAGTAAGGATTGACAGACTGAGAGCTCTT

M169_Lawr_virido-grisea TCAACACGGGGAAACTTACCAGGTCCAGACATAGTAAGGATTGACAGACTGAGAGCTCTT

M172_Lawr_glomerata TCAACACGGGGAAACTTACCAGGTCCAGACATAGTAAGGATTGACAGACTGAGAGCTCTT

M179_Lawr_glomerata TCAACACGGGGAAACTTACCAGGTCCAGACATAGTAAGGATTGACAGACTGAGAGCTCTT

M182_Lawr_helmsii TCAACACGGGGAAACTTACCAGGTCCAGACATAGTAAGGATTGACAGACTGAGAGCTCTT

M176_Lawr_berthae TCAACACGGGGAAACTTACCAGGTCCAGACATAGTAAGGATTGACAGACTGAGAGCTCTT

M183_Sida_hookeriana TCAACACGGGGAAACTTACCAGGTCCAGACATAGTAAGGATTGACAGACTGAGAGCTCTT

M187_Lawr_diffusa TCAACACGGGGAAACTTACCAGGTCCAGACATAGTAAGGATTGACAGACTGAGAGCTCTT

M215_Ripa_hermaphrodita TCAACACGGGGAAACTTACCAGGTCCAGACATAGTAAGGATTGACAGACTGAGAGCTCTT

M202_Hoheria_equitum TCAACACGGGGAAACTTACCAGGTCCAGACATAGTAAGGATTGACAGACTGAGAGCTCTT

M303_Hoheria_populnea TCAACACGGGGAAACTTACCAGGTCCAGACATAGTAAGGATTGACAGACTGAGAGCTCTT

1081 1090 1100 1110 1120 1130 1140

| | | | | | |

M196_Hoheria_populnea TCTTGATTCTATGGGTGGTGGTGCATGGCCGTTCTTAGTTGGTGGAGCGATTTGTCTGGT

M198_Hoheria_sextylosa TCTTGATTCTATGGGTGGTGGTGCATGGCCGTTCTTAGTTGGTGGAGCGATTTGTCTGGT

M199_Hoheria_angustifolia TCTTGATTCTATGGGTGGTGGTGCATGGCCGTTCTTAGTTGGTGGAGCGATTTGTCTGGT

M203_Hoheria_ovata TCTTGATTCTATGGGTGGTGGTGCATGGCCGTTCTTAGTTGGTGGAGCGATTTGTCTGGT

M207_Plagianthus_regius TCTTGATTCTATGGGTGGTGGTGCATGGCCGTTCTTAGTTGGTGGAGCGATTTGTCTGGT

M197_Plag_divaricatus TCTTGATTCTATGGGTGGTGGTGCATGGCCGTTCTTAGTTGGTGGAGCGATTTGTCTGGT

M206_Asterotrichion_discolor TCTTGATTCTATGGGTGGTGGTGCATGGCCGTTCTTAGTTGGTGGAGCGATTTGTCTGGT

M211_Asterotrichion_discolor TCTTGATTCTATGGGTGGTGGTGCATGGCCGTTCTTAGTTGGTGGAGCGATTTGTCTGGT

M212_Gynatrix_pulchella TCTTGATTCTATGGGTGGTGGTGCATGGCCGTTCTTAGTTGGTGGAGCGATTTGTCTGGT

M30_L_chubutensis TCTTGATTCTATGGGTGGTGGTGCATGGCCGTTCTTAGTTGGTGGAGCGATTTGTCTGGT

M168_Lawr_squamata TCTTGATTCTATGGGTGGTGGTGCATGGCCGTTCTTAGTTGGTGGAGCGATTTGTCTGGT

M169_Lawr_virido-grisea TCTTGATTCTATGGGTGGTGGTGCATGGCCGTTCTTAGTTGGTGGAGCGATTTGTCTGGT

M172_Lawr_glomerata TCTTGATTCTATGGGTGGTGGTGCATGGCCGTTCTTAGTTGGTGGAGCGATTTGTCTGGT

M179_Lawr_glomerata TCTTGATTCTATGGGTGGTGGTGCATGGCCGTTCTTAGTTGGTGGAGCGATTTGTCTGGT

M182_Lawr_helmsii TCTTGATTCTATGGGTGGTGGTGCATGGCCGTTCTTAGTTGGTGGAGCGATTTGTCTGGT

M176_Lawr_berthae TCTTGATTCTATGGGTGGTGGTGCATGGCCGTTCTTAGTTGGTGGAGCGATTTGTCTGGT

M183_Sida_hookeriana TCTTGATTCTATGGGTGGTGGTGCATGGCCGTTCTTAGTTGGTGGAGCGATTTGTCTGGT

M187_Lawr_diffusa TCTTGATTCTATGGGTGGTGGTGCATGGCCGTTCTTAGTTGGTGGAGCGATTTGTCTGGT

M215_Ripa_hermaphrodita TCTTGATTCTATGGGTGGTGGTGCATGGCCGTTCTTAGTTGGTGGAGCGATTTGTCTGGT

M202_Hoheria_equitum TCTTGATTCTATGGGTGGTGGTGCATGGCCGTTCTTAGTTGGTGGAGCGATTTGTCTGGT

M303_Hoheria_populnea TCTTGATTCTATGGGTGGTGGTGCATGGCCGTTCTTAGTTGGTGGAGCGATTTGTCTGGT

1141 1150 1160 1170 1180 1190 1200

| | | | | | |

M196_Hoheria_populnea TAATTCCGTTAACGAACGAGACCTCAGCCTGCTAACTAGCTACACGGAGGTGATCCTCCG

M198_Hoheria_sextylosa TAATTCCGTTAACGAACGAGACCTCAGCCTGCTAACTAGCTACACGGAGGTGATCCTCCG

M199_Hoheria_angustifolia TAATTCCGTTAACGAACGAGACCTCAGCCTGCTAACTAGCTACACGGAGGTGATCCTCCG

M203_Hoheria_ovata TAATTCCGTTAACGAACGAGACCTCAGCCTGCTAACTAGCTACACGGAGGTGATCCTCCG

M207_Plagianthus_regius TAATTCCGTTAACGAACGAGACCTCAGCCTGCTAACTAGCTACACGGAGGTGATCCTCCG

M197_Plag_divaricatus TAATTCCGTTAACGAACGAGACCTCAGCCTGCTAACTAGCTACACGGAGGTGATCCTCCG

M206_Asterotrichion_discolor TAATTCCGTTAACGAACGAGACCTCAGCCTGCTAACTAGCTACACGGAGGTGATCCTCCG

M211_Asterotrichion_discolor TAATTCCGTTAACGAACGAGACCTCAGCCTGCTAACTAGCTACACGGAGGTGATCCTCCG

M212_Gynatrix_pulchella TAATTCCGTTAACGAACGAGACCTCAGCCTGCTAACTAGCTACGCGGAGGTGATCCTCCG

M30_L_chubutensis TAATTCCGTTAACGAACGAGACCTCAGCCTGCTAACTAGCTACACGGAGGTGATCCTCCG

M168_Lawr_squamata TAATTCCGTTAACGAACGAGACCTCAGCCTGCTAACTAGCTACACGGAGGTGATCCTCCG

M169_Lawr_virido-grisea TAATTCCGTTAACGAACGAGACCTCAGCCTGCTAACTAGCTACACGGAGGTGATCCTCCG

M172_Lawr_glomerata TAATTCCGTTAACGAACGAGACCTCAGCCTGCTAACTAGCTACACGGAGGTGATCCTCCG

M179_Lawr_glomerata TAATTCCGTTAACGAACGAGACCTCAGCCTGCTAACTAGCTACACGGAGGTGATCCTCCG

M182_Lawr_helmsii TAATTCCGTTAACGAACGAGACCTCAGCCTGCTAACTAGCTACACGGAGGTGATCCTCCG

M176_Lawr_berthae TAATTCCGTTAACGAACGAGACCTCAGCCTGCTAACTAGCTACACGGAGGTGATCCTCCG

M183_Sida_hookeriana TAATTCCGTTAACGAACGAGACCTCAGCCTGCTAACTAGCTACACGGAGGTGATCCTCCG

M187_Lawr_diffusa TAATTCCGTTAACGAACGAGACCTCAGCCTGCTAACTAGCTACACGGAGGTGATCCTCCG

M215_Ripa_hermaphrodita TAATTCCGTTAACGAACGAGACCTCAGCCTGCTAACTAGCTACACGGAGGTGATCCTCCG

M202_Hoheria_equitum TAATTCCGTTAACGAACGAGACCTCAGCCTGCTAACTAGCTACACGGAGGTGATCCTCCG

M303_Hoheria_populnea TAATTCCGTTAACGAACGAGACCTCAGCCTGCTAACTAGCTACACGGAGGTGATCCTCCG

*

1201 1210 1220 1230 1240 1250 1260

| | | | | | |

M196_Hoheria_populnea TGGCTAGCTTCTTAGAGGGACTATGGCCGCTTAGGCCAAGGAAGTTTGAGGCAATAACAG

M198_Hoheria_sextylosa TGGCTAGCTTCTTAGAGGGACTATGGCCGCTTAGGCCAAGGAAGTTTGAGGCAATAACAG

M199_Hoheria_angustifolia TGGCTAGCTTCTTAGAGGGACTATGGCCGCTTAGGCCAAGGAAGTTTGAGGCAATAACAG

M203_Hoheria_ovata TGGCTAGCTTCTTAGAGGGACTATGGCCGCTTAGGCCAAGGAAGTTTGAGGCAATAACAG

M207_Plagianthus_regius TGGCTAGCTTCTTAGAGGGACTATGGCCGCTTAGGCCAAGGAAGTTTGAGGCAATAACAG

M197_Plag_divaricatus TGGCTAGCTTCTTAGAGGGACTATGGCCGCTTAGGCCAAGGAAGTTTGAGGCAATAACAG

M206_Asterotrichion_discolor TGGCTAGCTTCTTAGAGGGACTATGGCCGCTTAGGCCAAGGAAGTTTGAGGCAATAACAG

M211_Asterotrichion_discolor TGGCTAGCTTCTTAGAGGGACTATGGCCGCTTAGGCCAAGGAAGTTTGAGGCAATAACAG

M212_Gynatrix_pulchella TGGCTAGCTTCTTAGAGGGACTATGGCCGCTTAGGCCAAGGAAGTTTGAGGCAATAACAG

M30_L_chubutensis TGGCTAGCTTCTTAGAGGGACTATGGCCGCTTAGGCCAAGGAAGTTTGAGGCAATAACAG

M168_Lawr_squamata TGGCTAGCTTCTTAGAGGGACTATGGCCGCTTAGGCCAAGGAAGTTTGAGGCAATAACAG

M169_Lawr_virido-grisea TGGCTAGCTTCTTAGAGGGACTATGGCCGCTTAGGCCAAGGAAGTTTGAGGCAATAACAG

M172_Lawr_glomerata TGGCTAGCTTCTTAGAGGGACTATGGCCGCTTAGGCCAAGGAAGTTTGAGGCAATAACAG

M179_Lawr_glomerata TGGCTAGCTTCTTAGAGGGACTATGGCCGCTTAGGCCAAGGAAGTTTGAGGCAATAACAG

M182_Lawr_helmsii TGGCTAGCTTCTTAGAGGGACTATGGCCGCTTAGGCCAAGGAAGTTTGAGGCAATAACAG

M176_Lawr_berthae TGGCTAGCTTCTTAGAGGGACTATGGCCGCTTAGGCCAAGGAAGTTTGAGGCAATAACAG

M183_Sida_hookeriana TGGCTAGCTTCTTAGAGGGACTATGGCCGCTTAGGCCAAGGAAGTTTGAGGCAATAACAG

M187_Lawr_diffusa TGGCTAGCTTCTTAGAGGGACTATGGCCGCTTAGGCCAAGGAAGTTTGAGGCAATAACAG

M215_Ripa_hermaphrodita TGGCTAGCTTCTTAGAGGGACTATGGCCGCTTAGGCCAAGGAAGTTTGAGGCAATAACAG

M202_Hoheria_equitum TGGCTAGCTTCTTAGAGGGACTATGGCCGCTTAGGCCAAGGAAGTTTGAGGCAATAACAG

M303_Hoheria_populnea TGGCTAGCTTCTTAGAGGGACTATGGCCGCTTAGGCCAAGGAAGTTTGAGGCAATAACAG

1261 1270 1280 1290 1300 1310 1320

| | | | | | |

M196_Hoheria_populnea GTCTGTGATGCCCTTAGATGTTCTGGGCCGCACGCGCGCTACACTGATGTATTCAACGAG

M198_Hoheria_sextylosa GTCTGTGATGCCCTTAGATGTTCTGGGCCGCACGCGCGCTACACTGATGTATTCAACGAG

M199_Hoheria_angustifolia GTCTGTGATGCCCTTAGATGTTCTGGGCCGCACGCGCGCTACACTGATGTATTCAACGAG

M203_Hoheria_ovata GTCTGTGATGCCCTTAGATGTTCTGGGCCGCACGCGCGCTACACTGATGTATTCAACGAG

M207_Plagianthus_regius GTCTGTGATGCCCTTAGATGTTCTGGGCCGCACGCGCGCTACACTGATGTATTCAACGAG

M197_Plag_divaricatus GTCTGTGATGCCCTTAGATGTTCTGGGCCGCACGCGCGCTACACTGATGTATTCAACGAG

M206_Asterotrichion_discolor GTCTGTGATGCCCTTAGATGTTCTGGGCCGCACGCGCGCTACACTGATGTATTCAACGAG

M211_Asterotrichion_discolor GTCTGTGATGCCCTTAGATGTTCTGGGCCGCACGCGCGCTACACTGATGTATTCAACGAG

M212_Gynatrix_pulchella GTCTGTGATGCCCTTAGATGTTCTGGGCCGCACGCGCGCTACACTGATGTATTCAACGAG

M30_L_chubutensis GTCTGTGATGCCCTTAGATGTTCTGGGCCGCACGCGCGCTACACTGATGTATTCAACGAG

M168_Lawr_squamata GTCTGTGATGCCCTTAGATGTTCTGGGCCGCACGCGCGCTACACTGATGTATTCAACGAG

M169_Lawr_virido-grisea GTCTGTGATGCCCTTAGATGTTCTGGGCCGCACGCGCGCTACACTGATGTATTCAACGAG

M172_Lawr_glomerata GTCTGTGATGCCCTTAGATGTTCTGGGCCGCACGCGCGCTACACTGATGTATTCAACGAG

M179_Lawr_glomerata GTCTGTGATGCCCTTAGATGTTCTGGGCCGCACGCGCGCTACACTGATGTATTCAACGAG

M182_Lawr_helmsii GTCTGTGATGCCCTTAGATGTTCTGGGCCGCACGCGCGCTACACTGATGTATTCAACGAG

M176_Lawr_berthae GTCTGTGATGCCCTTAGATGTTCTGGGCCGCACGCGCGCTACACTGATGTATTCAACGAG

M183_Sida_hookeriana GTCTGTGATGCCCTTAGATGTTCTGGGCCGCACGCGCGCTACACTGATGTATTCAACGAG

M187_Lawr_diffusa GTCTGTGATGCCCTTAGATGTTCTGGGCCGCACGCGCGCTACACTGATGTATTCAACGAG

M215_Ripa_hermaphrodita GTCTGTGATGCCCTTAGATGTTCTGGGCCGCACGCGCGCTACACTGATGTATTCAACGAG

M202_Hoheria_equitum GTCTGTGATGCCCTTAGATGTTCTGGGCCGCACGCGCGCTACACTGATGTATTCAACGAG

M303_Hoheria_populnea GTCTGTGATGCCCTTAGATGTTCTGGGCCGCACGCGCGCTACACTGATGTATTCAACGAG

1321 1330 1340 1350 1360 1370 1380

| | | | | | |

M196_Hoheria_populnea TCTATAGCCTTGGCCGACAGGCCCGGGTAATCTTTGAAATTTCATCGTGATGGGGATAGA

M198_Hoheria_sextylosa TCTATAGCCTTGGCCGACAGGCCCGGGTAATCTTTGAAATTTCATCGTGATGGGGATAGA

M199_Hoheria_angustifolia TCTATAGCCTTGGCCGACAGGCCCGGGTAATCTTTGAAATTTCATCGTGATGGGGATAGA

M203_Hoheria_ovata TCTATAGCCTTGGCCGACAGGCCCGGGTAATCTTTGAAATTTCATCGTGATGGGGATAGA

M207_Plagianthus_regius TCTATAGCCTTGGCCGACAGGCCCGGGTAATCTTTGAAATTTCATCGTGATGGGGATAGA

M197_Plag_divaricatus TCTATAGCCTTGGCCGACAGGCCCGGGTAATCTTTGAAATTTCATCGTGATGGGGATAGA

M206_Asterotrichion_discolor TCTATAGCCTTGGCCGACAGGCCCGGGTAATCTTTGAAATTTCATCGTGATGGGGATAGA

M211_Asterotrichion_discolor TCTATAGCCTTGGCCGACAGGCCCGGGTAATCTTTGAAATTTCATCGTGATGGGGATAGA

M212_Gynatrix_pulchella TCTATAGCCTTGGCCGACAGGCCCGGGTAATCTTTGAAATTTCATCGTGATGGGGATAGA

M30_L_chubutensis TCTATAGCCTTGGCCGACAGGCCCGGGTAATCTTTGAAATTTCATCGTGATGGGGATAGA

M168_Lawr_squamata TCTATAGCCTTGGCCGACAGGCCCGGGTAATCTTTGAAATTTCATCGTGATGGGGATAGA

M169_Lawr_virido-grisea TCTATAGCCTTGGCCGACAGGCCCGGGTAATCTTTGAAATTTCATCGTGATGGGGATAGA

M172_Lawr_glomerata TCTATAGCCTTGGCCGACAGGCCCGGGTAATCTTTGAAATTTCATCGTGATGGGGATAGA

M179_Lawr_glomerata TCTATAGCCTTGGCCGACAGGCCCGGGTAATCTTTGAAATTTCATCGTGATGGGGATAGA

M182_Lawr_helmsii TCTATAGCCTTGGCCGACAGGCCCGGGTAATCTTTGAAATTTCATCGTGATGGGGATAGA

M176_Lawr_berthae TCTATAGCCTTGGCCGACAGGCCCGGGTAATCTTTGAAATTTCATCGTGATGGGGATAGA

M183_Sida_hookeriana TCTATAGCCTTGGCCGACAGGCCCGGGTAATCTTTGAAATTTCATCGTGATGGGGATAGA

M187_Lawr_diffusa TCTATAGCCTTGGCCGACAGGCCCGGGTAATCTTTGAAATTTCATCGTGATGGGGATAGA

M215_Ripa_hermaphrodita TCTATAGCCTTGGCCGACAGGCCCGGGTAATCTTTGAAATTTCATCGTGATGGGGATAGA

M202_Hoheria_equitum TCTATAGCCTTGGCCGACAGGCCCGGGTAATCTTTGAAATTTCATCGTGATGGGGATAGA

M303_Hoheria_populnea TCTATAGCCTTGGCCGACAGGCCCGGGTAATCTTTGAAATTTCATCGTGATGGGGATAGA

1381 1390 1400 1410 1420 1430 1440

| | | | | | |

M196_Hoheria_populnea TCATTGCAATTGTTGGTCTTCAACGAGGAATTCCTAGTAAGCGCGAGTCATCAGCTCGCG

M198_Hoheria_sextylosa TCATTGCAATTGTTGGTCTTCAACGAGGAATTCCTAGTAAGCGCGAGTCATCAGCTCGCG

M199_Hoheria_angustifolia TCATTGCAATTGTTGGTCTTCAACGAGGAATTCCTAGTAAGCGCGAGTCATCAGCTCGCG

M203_Hoheria_ovata TCATTGCAATTGTTGGTCTTCAACGAGGAATTCCTAGTAAGCGCGAGTCATCAGCTCGCG

M207_Plagianthus_regius TCATTGCAATTGTTGGTCTTCAACGAGGAATTCCTAGTAAGCGCGAGTCATCAGCTCGCG

M197_Plag_divaricatus TCATTGCAATTGTTGGTCTTCAACGAGGAATTCCTAGTAAGCGCGAGTCATCAGCTCGCG

M206_Asterotrichion_discolor TCATTGCAATTGTTGGTCTTCAACGAGGAATTCCTAGTAAGCGCGAGTCATCAGCTCGCG

M211_Asterotrichion_discolor TCATTGCAATTGTTGGTCTTCAACGAGGAATTCCTAGTAAGCGCGAGTCATCAGCTCGCG

M212_Gynatrix_pulchella TCATTGCAATTGTTGGTCTTCAACGAGGAATTCCTAGTAAGCGCGAGTCATCAGCTCGCG

M30_L_chubutensis TCATTGCAATTGTTGGTCTTCAACGAGGAATTCCTAGTAAGCGCGAGTCATCAGCTCGCG

M168_Lawr_squamata TCATTGCAATTGTTGGTCTTCAACGAGGAATTCCTAGTAAGCGCGAGTCATCAGCTCGCG

M169_Lawr_virido-grisea TCATTGCAATTGTTGGTCTTCAACGAGGAATTCCTAGTAAGCGCGAGTCATCAGCTCGCG

M172_Lawr_glomerata TCATTGCAATTGTTGGTCTTCAACGAGGAATTCCTAGTAAGCGCGAGTCATCAGCTCGCG

M179_Lawr_glomerata TCATTGCAATTGTTGGTCTTCAACGAGGAATTCCTAGTAAGCGCGAGTCATCAGCTCGCG

M182_Lawr_helmsii TCATTGCAATTGTTGGTCTTCAACGAGGAATTCCTAGTAAGCGCGAGTCATCAGCTCGCG

M176_Lawr_berthae TCATTGCAATTGTTGGTCTTCAACGAGGAATTCCTAGTAAGCGCGAGTCATCAGCTCGCG

M183_Sida_hookeriana TCATTGCAATTGTTGGTCTTCAACGAGGAATTCCTAGTAAGCGCGAGTCATCAGCTCGCG

M187_Lawr_diffusa TCATTGCAATTGTTGGTCTTCAACGAGGAATTCCTAGTAAGCGCGAGTCATCAGCTCGCG

M215_Ripa_hermaphrodita TCATTGCAATTGTTGGTCTTCAACGAGGAATTCCTAGTAAGCGCGAGTCATCAGCTCGCG

M202_Hoheria_equitum TCATTGCAATTGTTGGTCTTCAACGAGGAATTCCTAGTAAGCGCGAGTCATCAGCTCGCG

M303_Hoheria_populnea TCATTGCAATTGTTGGTCTTCAACGAGGAATTCCTAGTAAGCGCGAGTCATCAGCTCGCG

1441 1450 1460 1470 1480 1490 1500

| | | | | | |

M196_Hoheria_populnea TTGACTACGTCCCTGCCCTTTGTACACACCGCCCGTCGCTCCTACCGATTGAATGGTCCG

M198_Hoheria_sextylosa TTGACTACGTCCCTGCCCTTTGTACACACCGCCCGTCGCTCCTACCGATTGAATGGTCCG

M199_Hoheria_angustifolia TTGACTACGTCCCTGCCCTTTGTACACACCGCCCGTCGCTCCTACCGATTGAATGGTCCG

M203_Hoheria_ovata TTGACTACGTCCCTGCCCTTTGTACACACCGCCCGTCGCTCCTACCGATTGAATGGTCCG

M207_Plagianthus_regius TTGACTACGTCCCTGCCCTTTGTACACACCGCCCGTCGCTCCTACCGATTGAATGGTCCG

M197_Plag_divaricatus TTGACTACGTCCCTGCCCTTTGTACACACCGCCCGTCGCTCCTACCGATTGAATGGTCCG

M206_Asterotrichion_discolor TTGACTACGTCCCTGCCCTTTGTACACACCGCCCGTCGCTCCTACCGATTGAATGGTCCG

M211_Asterotrichion_discolor TTGACTACGTCCCTGCCCTTTGTACACACCGCCCGTCGCTCCTACCGATTGAATGGTCCG

M212_Gynatrix_pulchella TTGACTACGTCCCTGCCCTTTGTACACACCGCCCGTCGCTCCTACCGATTGAATGGTCCG

M30_L_chubutensis TTGACTACGTCCCTGCCCTTTGTACACACCGCCCGTCGCTCCTACCGATTGAATGGTCCG

M168_Lawr_squamata TTGACTACGTCCCTGCCCTTTGTACACACCGCCCGTCGCTCCTACCGATTGAATGGTCCG

M169_Lawr_virido-grisea TTGACTACGTCCCTGCCCTTTGTACACACCGCCCGTCGCTCCTACCGATTGAATGGTCCG

M172_Lawr_glomerata TTGACTACGTCCCTGCCCTTTGTACACACCGCCCGTCGCTCCTACCGATTGAATGGTCCG

M179_Lawr_glomerata TTGACTACGTCCCTGCCCTTTGTACACACCGCCCGTCGCTCCTACCGATTGAATGGTCCG

M182_Lawr_helmsii TTGACTACGTCCCTGCCCTTTGTACACACCGCCCGTCGCTCCTACCGATTGAATGGTCCG

M176_Lawr_berthae TTGACTACGTCCCTGCCCTTTGTACACACCGCCCGTCGCTCCTACCGATTGAATGGTCCG

M183_Sida_hookeriana TTGACTACGTCCCTGCCCTTTGTACACACCGCCCGTCGCTCCTACCGATTGAATGGTCCG

M187_Lawr_diffusa TTGACTACGTCCCTGCCCTTTGTACACACCGCCCGTCGCTCCTACCGATTGAATGGTCCG

M215_Ripa_hermaphrodita TTGACTACGTCCCTGCCCTTTGTACACACCGCCCGTCGCTCCTACCGATTGAATGGTCCG

M202_Hoheria_equitum TTGACTACGTCCCTGCCCTTTGTACACACCGCCCGTCGCTCCTACCGATTGAATGGTCCG

M303_Hoheria_populnea TTGACTACGTCCCTGCCCTTTGTACACACCGCCCGTCGCTCCTACCGATTGAATGGTCCG

1501 1510 1520 1530 1540 1550 1560

| | | | | | |

M196_Hoheria_populnea GTGAAATGTTCGGATCGCGGCGACGTGGGCGGTTCGCTGCCCGCGACGTCGCGAGAAGTC

M198_Hoheria_sextylosa GTGAAATGTTCGGATCGCGGCGACGTGGGCGGTTCGCTGCCCGCGACGTCGCGAGAAGTC

M199_Hoheria_angustifolia GTGAAATGTTCGGATCGCGGCGACGTGGGCGGTTCGCTGCCCGCGACGTCGCGAGAAGTC

M203_Hoheria_ovata GTGAAATGTTCGGATCGCGGCGACGTGGGCGGTTCGCTGCCCGCGACGTCGCGAGAAGTC

M207_Plagianthus_regius GTGAAATGTTCGGATCGCGGCGACGTGGGCGGTTCGCTGCCCGCGACGTCGCGAGAAGTC

M197_Plag_divaricatus GTGAAATGTTCGGATCGCGGCGACGTGGGCGGTTCGCTGCCCGCGACGTCGCGAGAAGTC

M206_Asterotrichion_discolor GTGAAATGTTCGGATCGCGGCGACGTGGGCGGTTCGCTGCCCGCGACGTCGCGAGAAGTC

M211_Asterotrichion_discolor GTGAAATGTTCGGATCGCGGCGACGTGGGCGGTTCGCTGCCCGCGACGTCGCGAGAAGTC

M212_Gynatrix_pulchella GTGAAATGTTCGGATCGCGGCGACGTGGGCGGTTCGCTGCCCGCGACGTCGCGAGAAGTC

M30_L_chubutensis GTGAAATGTTCGGATCGCGGCGACGTGGGCGGTTCGCTGCCCGCGACGTCGCGAGAAGTC

M168_Lawr_squamata GTGAAATGTTCGGATCGCGGCGACGTGGGCGGTTCGCTGCCCGCGACGTCGCGAGAAGTC

M169_Lawr_virido-grisea GTGAAATGTTCGGATCGCGGCGACGTGGGCGGTTCGCTGCCCGCGACGTCGCGAGAAGTC

M172_Lawr_glomerata GTGAAATGTTCGGATCGCGGCGACGTGGGCGGTTCGCTGCCCGCGACGTCGCGAGAAGTC

M179_Lawr_glomerata GTGAAATGTTCGGATCGCGGCGACGTGGGCGGTTCGCTGGCCGCGACGTCGCGAGAAGTC

M182_Lawr_helmsii GTGAAATGTTCGGATCGCGGCGACGTGGGCGGTTCGCTGCCCGCGACGTCGCGAGAAGTC

M176_Lawr_berthae GTGAAATGTTCGGATCGCGGCGACGTGGGCGGTTCGCTGCCCGCGACGTCGCGAGAAGTC

M183_Sida_hookeriana GTGAAATGTTCGGATCGCGGCGACGTGGGCGGTTCGCTGCCCGCGACGTCGCGAGAAGTC

M187_Lawr_diffusa GTGAAATGTTCGGATCGCGGCGACGTGGGCGGTTCGCTGCCCGCGACGTCGCGAGAAGTC

M215_Ripa_hermaphrodita GTGAAATGTTCGGATCGCGGCGACGTGGGCGGTTCGCTGCCCGCGACGTCGCGAGAAGTC

M202_Hoheria_equitum GTGAAATGTTCGGATCGCGGCGACGTGGGCGGTTCGCTGCCCGCGACGTCGCGAGAAGTC

M303_Hoheria_populnea GTGAAATGTTCGGATCGCGGCGACGTGGGCGGTTCGCTGCCCGCGACGTCGCGAGAAGTC

*

1561 1570 1580 1590

| | | |

M196_Hoheria_populnea CATTGAACCTTATCATTTAGAGGAAGGAGAA

M198_Hoheria_sextylosa CATTGAACCTTATCATTTAGAGGAAGGAGA-

M199_Hoheria_angustifolia CATTGAACCTTATCATTTAGAGGAAGGAGAA

M203_Hoheria_ovata CATTGAACCTTATCATTTAGAGGAAGGAGAA

M207_Plagianthus_regius CATTGAACCTTATCATTTAGAGGAAGGAGAA

M197_Plag_divaricatus CATTGAACCTTATCATTTAGAGGAAGGAGAA

M206_Asterotrichion_discolor CATTGAACCTTATCATTTAGAGGAAGGAGA-

M211_Asterotrichion_discolor CATTGAACCTTATCATTTAGAGGAAGGAGAA

M212_Gynatrix_pulchella CATTGAACCTTATCATTTAGAGGAAGG----

M30_L_chubutensis CATTGAACCTTATCATTTAGAGGAAGGAGAA

M168_Lawr_squamata CATTGAACCTTATCATTTAGAGGAAGGAGAA

M169_Lawr_virido-grisea CATTGAACCTTATCATTTAGAGGAAGGAGAA

M172_Lawr_glomerata CATTGAACCTTATCATTTAGAGGAAGGAGAA

M179_Lawr_glomerata CATTGAACCTTATCATTTAGAGGAAGGAGAA

M182_Lawr_helmsii CATTGAACCTTATCATTTAGAGGAAGGAGAA

M176_Lawr_berthae CATTGAACCTTATCATTTAGAGGAAGGAGAA

M183_Sida_hookeriana CATTGAACCTTATCATTTAGAGGAAGGAGAA

M187_Lawr_diffusa CATTGAACCTTATCATTTAGAGGAAGGAGAA

M215_Ripa_hermaphrodita CATTGAACCTTATCATTTAGAGGAAGGAGAA

M202_Hoheria_equitum CATTGAACCTTATCATTTAGAGGAAGGAGAA

M303_Hoheria_populnea CATTGAACCTTATCATTTAGAGGAAGGAG--
