# Supplementary material for: Polyploidy on islands – concerted evolution and gene loss amid chromosomal stasis
Source: Ann Bot. 2022 Apr 7;131(1):33–44. doi: 10.1093/aob/mcac051 (PMC9904340; doi:10.1093/aob/mcac051)
Supplement: mcac051_suppl_Supplementary_Table_S1 [file mcac051_suppl_supplementary_table_s1.docx]

Supplementary Table 1. Voucher information for species used for cytogenetic (C) and phylogenetic (P) study and GenBank accession numbers for newly generated sequences.

| Taxon | DNA code | Use | Voucher | Locality | 18S | ITS | *GBSSI-1* |
| --- | --- | --- | --- | --- | --- | --- | --- |
| *Asterotrichion discolor* | M206 | P | Jordan s.n. (HO) | Australia, Tasmania | MZ947189 | MZ947218 | 1A- MZ964626 |
| *Asterotrichion discolor* | M211 | P | Jordan s.n. (HO) | Australia, Tasmania | MZ947190 | MZ947220 | 1A- MZ964627  1B- MZ964628 |
| *Asterotrichion discolor* | M306 | C/P | Tate 124 (MPN) | New Zealand^1^ | OK001714 | -- | 1A- MZ964629  1B- MZ964630 |
| *Gynatrix pulchella* | M212 | P | Purdie 4912A (CANB) | Australia | MZ947191 | MZ947221 | 1A- MZ964631  1B- MZ964632 |
| *Hoheria angustifolia* | M199 | C/P | Skema 499 (MPN) | New Zealand | MZ947185 | MZ947215 | 1A - MZ964633  1B - MZ964634  1C - MZ964635 |
| *Hoheria equitum* | M202 | P | de Lange 6544 (AKL) | New Zealand | MZ947202 | MZ947216 | 1A - MZ964636  1B - MZ964637  **1C -** MZ964638 |
| *Hoheria ovata* | M203 | P | de Lange 6546 (CHR) | New Zealand | MZ947186 | MZ947217 | 1A - MZ964639  1B - MZ964640  1C - MZ964641 |
| *Hoheria populnea* | M196 | P | de Lange 9750 (AKL) | New Zealand | MZ947183 | MZ947223 | 1A - MZ964642  1B - MZ964643 |
| *Hoheria populnea* | M303 | C/P | Tate 123 (MPN) | New Zealand | MZ947203 | MZ947224 | 1A - MZ964644  1C - MZ964645 |
| *Hoheria sexstylosa* | M198 | P | Skema 505 (MPN) | New Zealand | MZ947184 | MZ947214 | 1B - MZ964646  1C - MZ964647 |
| *Lawrencia berthae* | M176 | P | Barker 8711 (AD) | Australia | MZ947198 | MZ947208 | 1A - MZ964648  1B - MZ964649  1C - MZ964650 |
| *Lawrencia diffusa* | M187 | P | Eichler 20317 (AD) | Australia | MZ947200 | MZ947212 | 1C - MZ964651 |
| *Lawencia glomerata* | M172 | P | Barker 7411 (AD) | Australia | MZ947195 | MZ947207 | 1C - MZ964652 |
| *Lawrencia glomerata* | M179 | P | Barker 7481 (AD) | Australia | MZ947196 | MZ947209 | 1C - MZ964653 |
| *Lawrencia helmsii* | M182 | P | Barker 7271 (AD) | Australia | MZ947197 | MZ947210 | 1C - MZ964654 |
| *Lawrencia squamata* | M168 | P | Barker 7482 (AD) | Australia | MZ947193 | MZ947205 | 1C - MZ964655 |
| *Lawrencia viridigrisea* | M169 | P | Barker 8657 (AD) | Australia | MZ947194 | MZ947206 | 1C - MZ964656 |
| *Lecanophora chubutensis* | M30 | P | Tate et al. 72 (TEX) | Argentina | MZ947192 | AY591837 | MZ964657 |
| *Plagianthus divaricatus* | M197 | C/P | Skema 479 (MPN) | New Zealand | MZ947188 | MZ947213 | 1A - MZ964658  1B - MZ964659 |
| *Plagianthus regius* | M207 | C/P | Skema 496 (MPN) | New Zealand | MZ947187 | MZ947219 | 1B - MZ964660 |
| *Ripariosida hermaphrodita* | M215 | P | Bradley & Kelly 6926 (GMU) | USA | MZ947201 | MZ947222 | MZ964661 |
| *Sida hookeriana* | M183 | P | Barker 8361 (AD) | Australia | MZ947199 | MZ947211 | 1B - MZ964662  1C - MZ964663 |

^1^grown from seed in New Zealand
